# The effect of travel restrictions on the geographical spread of COVID-19 between large cities in China: a modelling study

**DOI:** 10.1186/s12916-020-01712-9

**Published:** 2020-08-19

**Authors:** Billy J. Quilty, Charlie Diamond, Yang Liu, Hamish Gibbs, Timothy W. Russell, Christopher I. Jarvis, Kiesha Prem, Carl A. B. Pearson, Samuel Clifford, Stefan Flasche, CMMID COVID-19 working group, Jon C. Emery, Megan Auzenbergs, Nicholas Davies, Emily S. Nightingale, Kevin van Zandvoort, Thibaut Jombart, Arminder K. Deol, W. John Edmunds, Joel Hellewell, Sebastian Funk, Sam Abbott, Fiona Sun, Akira Endo, Alicia Rosello, Amy Gimma, Simon R. Procter, Nikos I. Bosse, Kathleen O’Reilly, Graham Medley, James D. Munday, Rein M. G. J. Houben, Adam J. Kucharski, Gwenan M. Knight, Petra Klepac, Rosalind M. Eggo, Mark Jit

**Affiliations:** Centre for Mathematical Modelling of Infectious Diseases, Department of Infectious Disease Epidemiology, London School of Hygiene and Tropical Medicine, Keppel Street, WC1E 7HT, London, UK

**Keywords:** Travel restrictions, COVID-19, Wuhan, China, Modelling, Outbreaks, Delay, SARS-CoV-2, Mobility, *Cordon sanitaire*

## Abstract

**Background:**

To contain the spread of COVID-19, a *cordon sanitaire* was put in place in Wuhan prior to the Lunar New Year, on 23 January 2020. We assess the efficacy of the *cordon sanitaire* to delay the introduction and onset of local transmission of COVID-19 in other major cities in mainland China.

**Methods:**

We estimated the number of infected travellers from Wuhan to other major cities in mainland China from November 2019 to February 2020 using previously estimated COVID-19 prevalence in Wuhan and publicly available mobility data. We focused on Beijing, Chongqing, Hangzhou, and Shenzhen as four representative major cities to identify the potential independent contribution of the *cordon sanitaire* and holiday travel. To do this, we simulated outbreaks generated by infected arrivals in these destination cities using stochastic branching processes. We also modelled the effect of the *cordon sanitaire* in combination with reduced transmissibility scenarios to simulate the effect of local non-pharmaceutical interventions.

**Results:**

We find that in the four cities, given the potentially high prevalence of COVID-19 in Wuhan between December 2019 and early January 2020, local transmission may have been seeded as early as 1–8 January 2020. By the time the *cordon sanitaire* was imposed, infections were likely in the thousands. The *cordon sanitaire* alone did not substantially affect the epidemic progression in these cities, although it may have had some effect in smaller cities. Reduced transmissibility resulted in a notable decrease in the incidence of infection in the four studied cities.

**Conclusions:**

Our results indicate that sustained transmission was likely occurring several weeks prior to the implementation of the *cordon sanitaire* in four major cities of mainland China and that the observed decrease in incidence was likely attributable to other non-pharmaceutical, transmission-reducing interventions.

## Background

Since late 2019, severe acute respiratory syndrome coronavirus 2 (SARS-CoV-2), the causative agent of coronavirus disease 2019 (COVID-19), has spread to over 114 countries and was declared a pandemic on 11 March 2020 [1]. Some countries have enacted *cordon sanitaire*-type travel restrictions, either to prevent the export of infections from an initial disease epicentre (such as Wuhan in January 2020 [2] or Northern Italy in March 2020 [3]) to other countries and regions or to prevent the import of infections from high-risk countries or regions (such as the USA’s ban on travel from Europe [4]). *Cordon sanitaires* aim to curb the number of infected travellers entering a region with a high proportion of susceptible individuals, where they may seed additional chains of transmission. However, historically, they at best delay, rather than prevent outbreaks elsewhere [5]. Hence, the efficacy of *cordon sanitaires* in averting or delaying outbreaks in other locations is an open question.

Chinese authorities imposed a *cordon sanitaire* on the city of Wuhan on 23 January 2020 [2] and extended the travel restrictions to the whole of Hubei province by 26 January 2020 [6]. The restrictions were imposed 1 day prior to the Lunar New Year (LNY) holidays and during *Chunyun*, the 40-day holiday travel period that marks the largest annual human migration event in the world [7]. At the same time, other public health interventions, such as physical distancing, were also enacted across China [8].

This study aims to assess the impacts of the *cordon sanitaire* around Wuhan, the epicentre of the COVID-19 pandemic, on reducing incidence and delaying outbreaks in other well-connected large population centres in mainland China. We used publicly available mobility data based on location-based service (LBS) provided by Baidu Huiyan, to construct four mobility scenarios. Combined with daily estimated prevalence of COVID-19 in Wuhan before 11 February 2020 by Kucharski et al. [9], we simulated the daily importations of infected travellers to Beijing, Chongqing, Hangzhou, and Shenzhen to assess the risk that they would cause sustained local transmission.

## Methods

### Estimating number of infected travellers

We obtained daily prefecture-level human mobility data, expressed by a relative index scale, for mainland China from Baidu Huiyan for both the 2019 and 2020 travel periods surrounding the LNY, known as *Chunyun*. The platform aggregates mobile phone travel data from an estimated 189 million daily active users, processing > 120 billion daily positioning requests mainly through WiFi and GPS [10].

We examined the proportions of the total outflow leaving Wuhan and entering all other prefectures in China (excluding Wuhan). We then selected Beijing, Chongqing, Hangzhou, and Shenzhen for further analysis as major population centres with substantial travel with Wuhan and a wide geographic spread. We assume that the early transmission dynamics of SARS-CoV-2 in cities of this size were similar to that in Wuhan.

To estimate the absolute number of daily travellers leaving Wuhan, we assumed that each unit of Baidu’s migration index corresponds linearly to 50,000 travellers. This was chosen as the most credible value after synthesising evidence from several sources [8, 11–14] (see Additional file 1: Supplementary Appendix 1).

We calculated the total number of daily travellers leaving Wuhan and entering each city by taking the product of the scaling factor, the total daily outflow index from Wuhan, and the daily proportion of travellers from Wuhan entering the four cities. Daily estimated COVID-19 prevalence in Wuhan was retrieved from the exposed (incubating) and infectious compartments of a published SEIR model on the early dynamics of COVID-19 transmission in Wuhan [9]. We estimated the number of daily infected arrivals in a destination city as a Poisson process governed by the daily number of travellers and prevalence in Wuhan (Additional file 1: Supplementary Appendix 2). Each day, we simulated this arrival process 100 times to capture the uncertainty in the process; this represents 7100 samples for the 71 days for each city in each scenario. We assumed that individuals would travel regardless of their infection status, and Wuhan was the sole source of infected individuals and populations within destination cities mixed homogeneously.

We examined four travel scenarios (Table 1): Scenario 1 is based on the observed travel pattern in 2020 and represents the *Chunyun* period with *cordon sanitaire* introduced on 23 January. Scenario 2 represents a counterfactual travel pattern used to evaluate how the COVID-19 outbreak would spread if no *cordon sanitaire* was implemented. This was based upon the actual travel from Wuhan for the equivalent *Chunyun* period in 2019. In scenario 3, we synthesised a hypothetical travel pattern to represent a typical non-*Chunyun* period with *cordon sanitaire* introduced on 23 January, using outward travel flow on representative non-*Chunyun* days in 2019. Scenario 4 is a variation on scenario 3 in which no *cordon sanitaire* was implemented.

**Table 1:** Scenarios describing different possible travel patterns out of Wuhan used in simulations

Scenario	Time of the year	Source year	*Cordon sanitaire* imposed	Observed/hypothetical
1	*Chunyun*	2020	Yes	Observed
2	*Chunyun*	2019	No	Observed
3	Non-*Chunyun*	2019 and 2020	Yes	Hypothetical
4	Non-*Chunyun*	2019	No	Hypothetical

We extended the corresponding outflow time series to the early stages of the outbreak (22 November 2019), by assuming the outflow from Wuhan to equal to the average daily outflow on representative non-*Chunyun* days, whilst accounting for weekday effects. The pairwise travel flow proportions between Wuhan and each other prefecture-level city was only available between 1 January and 1 March, 2020, so an approximation of the general flow magnitude was used for dates outside of the observed range (22 November–31 December) and in simulated aspects of our scenarios, i.e. *Chunyun* affected travel days in non-*Chunyun* scenarios. A more detailed description of how each scenario was formulated is in Additional file 1: Supplementary Appendix 3.

### Branching process transmission model

As cases in China during the early epidemic were likely underreported [15], we used a stochastic branching process model to simulate outbreaks in each of the four cities. Consistent with the prevalence estimates from Wuhan [9], we began simulating travel from Wuhan on 22 November 2019 and calculated incidence up to 1 February 2020. For each simulated infected arrival in each city on a given day, an independent branching process is generated, with:

- A negative binomial offspring distribution with a time-varying mean effective reproduction number (*R*_*e*_) with baseline 2.2 [16] and overdispersion (*k*, variability in the number of secondary cases resulting from an infected case*)* of 0.1 [17]
- A log-normal serial interval (SI) with mean of 4.7 days and standard deviation of 2.9 [18]

We assume that in the initial phases of the epidemic (prior to the *cordon sanitaire*), the effective daily reproduction number (*R*_*e*_) was 2.2 [16, 19]. The date at which the probability of sustained transmission exceeded a threshold of 95% (i.e. an outbreak occurring) given *R*_*e*_ of 2.2 and *k* = 0.1 was used to evaluate the effect of travel restrictions (details in Additional file 1: Supplementary Appendix 4) [20]. A sensitivity analysis for *k* using the lower and upper bounds from Endo et al. [17] (0.04, 0.2) and H1N1-like (2.0) [21] overdispersion in *R*_*e*_ is shown in Fig. 4. We also perform a sensitivity analysis on the serial interval, using a gamma-distributed SI of mean 7.5 days and standard deviation of 3.4 days [19] (Additional file 1: Figure S3 and S5) [18, 22]. To simulate the effect of local non-pharmaceutical intervention measures (NPIs) such as physical distancing and workplace and school closures in addition to travel restrictions [23], we compare *R*_*e*_ = 2.2 in the absence of interventions (no change, unmitigated local outbreak), to 1.1 (50% reduction, slowing epidemic, *R*_*e*_ > 1), or 0.55 (75% reduction, suppressing epidemic, *R*_*e*_ < 1). We assume additional interventions took effect on the same date as the introduction of the *cordon sanitaire*, 23 January 2020.

### Implementation

All analyses were carried out using R version 3.6.2. The branching process model was implemented using the package *projections* version 0.4.1 [24].

## Results

### Effect of the *cordon sanitaire* on mobility

**Fig. 1:**
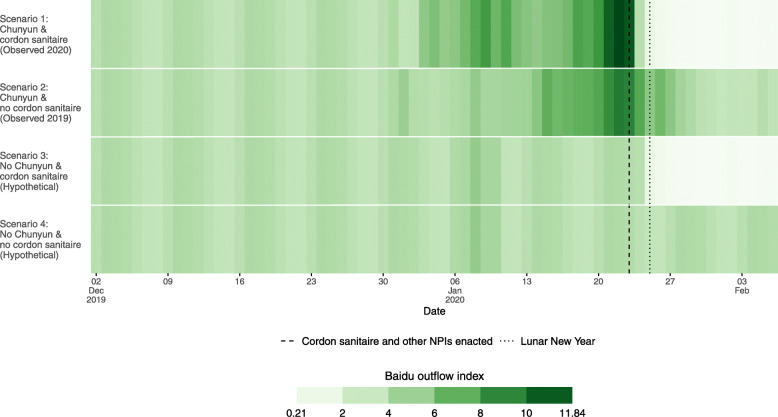
Total domestic travel outflow from Wuhan under 4 travel pattern scenarios

A gradual increase in the outflow from Wuhan in the weeks prior to the LNY was observed in both 2020 and 2019, exemplifying the *Chunyun* period (Fig. 1). Comparing the 23 days prior to the introduction of the *cordon sanitaire* in scenarios 1 and 2, we estimate daily outflow was 21.7% (95% CI 9.78–33.6%) higher in 2020 than the equivalent period in 2019. A surge in volume in the 3 days preceding the *cordon sanitaire* can be seen in scenario 1 (2020), where an estimated 1.69 million left Wuhan, in line with other estimates [8]. A similar outflow immediately before the LNY observed in scenario 2 (2019) suggests the surge cannot necessarily be attributed to upcoming travel restrictions. This is further reflected by the 22.5% between-year increase during this 3-day window not being substantially greater than the average daily outflow increase.

The *cordon sanitaire* had a stark effect on reducing the total outflow from Wuhan. Comparing the mean daily outflow in the 23 days preceding restrictions with the 23 days after, volume fell by 92.7%, from 345,000 (95% CI 299,000–390,000) average daily travellers to 25,300 (95% CI 8590–42,000). In comparison, volume fell by 30.2% during the equivalent period in 2019 from 290,000 (95% CI 252,000–328,000) to 203,000 (95% CI 177,000–228,000). After restrictions were imposed, travel volume declined to a low plateau over 5 days, during which approximately 330,000 people left. On the lowest day (3 February), we estimate 10,500 people left Wuhan, which likely represents only essential journeys.

In our hypothetical scenarios, we simulated the outbound flow with the additional travel volume due to *Chunyun* removed. By comparing scenarios 2 and 4 during *Chunyun* (10 January–18 February, 2020) we estimate that 60,000 (95% CI 32,000–88,100) extra travellers left Wuhan every day because of *Chunyun*.

We found that in all but one prefecture with over 7 million inhabitants, the *cordon sanitaire* on 23 January did not substantially change the time at which sustained transmission was likely to occur (Additional file 1: Figure S1 A-F), but the picture was more mixed in smaller cities. Of the four representative major cities selected for further analysis, during their pre-restriction travel phase in scenario 1 (1 January–23 January, 2020): Beijing experienced a high volume of travel with approximately 1510 (95% CI 1200–1820) mean daily travellers from Wuhan; Chongqing had the highest at 1650 (95% CI 1320–1970); Hangzhou received relatively fewer with 451 (95% CI 362–541); and Shenzhen had a medium travel volume from Wuhan with 820 (95% CI 664–976) mean daily travellers.

### Effect of the *cordon sanitaire* on importations of infected persons to other major Chinese cities

**Fig. 2:**
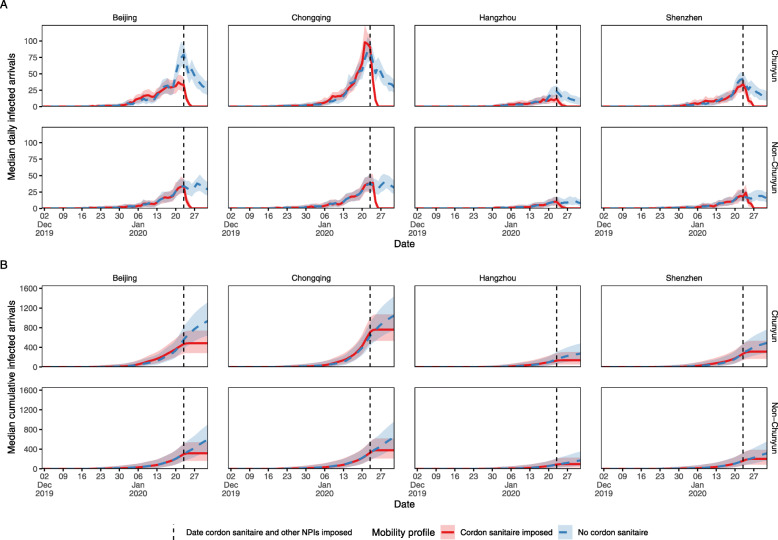
**a** Estimated median number of daily infected arrivals and **b** estimated cumulative number of infected arrivals from Wuhan for the four chosen cities (Beijing, Chongqing, Hangzhou, and Shenzhen, left to right) for *Chunyun* vs. non-*Chunyun* and *cordon sanitaire* imposed vs. no *cordon sanitaire*. The shaded area indicates the 95% uncertainty interval. The vertical dashed line indicates the date the *cordon sanitaire* was imposed

We estimate infected individuals began arriving on a daily basis in other major population centres in mid-December in scenario 1 (observed *Chunyun* travel profile and *cordon sanitaire imposed*) (Fig. 2). The estimated median number of infected arrivals on a given day peaked prior to the travel restrictions at 37 (95% uncertainty interval (UI) 26–47) in Beijing, 95 (95% UI 77–115) in Chongqing, 13 (95% UI 6–19) in Hangzhou, and 33 (95% UI 23–44) in Shenzhen. Travel restrictions reduced the number of infected arrivals to below 1 in all four cities within 2 days (Fig. 2a). In scenario 2 (*Chunyun* travel profile without *cordon sanitaire*), the number of daily infected arrivals decreases slightly after the *Chunyun* travel period (Fig. 2a). In cities with populations below 7 million, infected individuals began arriving later, so the *cordon sanitaire* may have acted to delay or prevent the arrival of infected individuals (Additional file 1: Figure S1 A-F).

In scenario 3 (non*-Chunyun* with travel restrictions), the estimated number of daily infected arrivals is marginally lower than scenario 1, peaking at 35 (95% UI 25–46) in Beijing, 39 (95% UI 28–50) in Chongqing, 11 (95% UI 5–17) in Hangzhou, and 25 (95% UI 15–34) in Shenzhen.

### Effect of the *cordon sanitaire* on outbreaks in other major Chinese cities

Due to the volume of outbound travel from Wuhan in scenario 1, we estimate that sustained local transmission was likely to have already occurred in the four cities in early January, several weeks prior to the introduction of the *cordon sanitaire* (Table 2). On the date travel restrictions from Wuhan were imposed, local infections were likely to be in the thousands in the four cities (Table 2). Outbreaks started later and were smaller on the date of the shutdown in Hangzhou and Shenzhen compared to Beijing and Chongqing, which reflects the relative volume of travel from Wuhan.

**Table 2:** Estimated number of local infections in each of the four cities of interest in the baseline scenario on 23 January 2020, the date the *cordon sanitaire* was imposed

Prefecture-level city	Cumulative number of infected arrivals by 23 January (median, 95% confidence interval)	Cumulative number of locally transmitted infections by 23 January (median, 95% uncertainty interval)
Beijing	465 (286–710)	4007 (1410–25,467)
Chongqing	713 (489–1007)	3936 (1321–29,678)
Hangzhou	127 (45–277)	1004 (229–12,030)
Shenzhen	271 (147–457)	1859 (399–14,261)

**Fig. 3:**
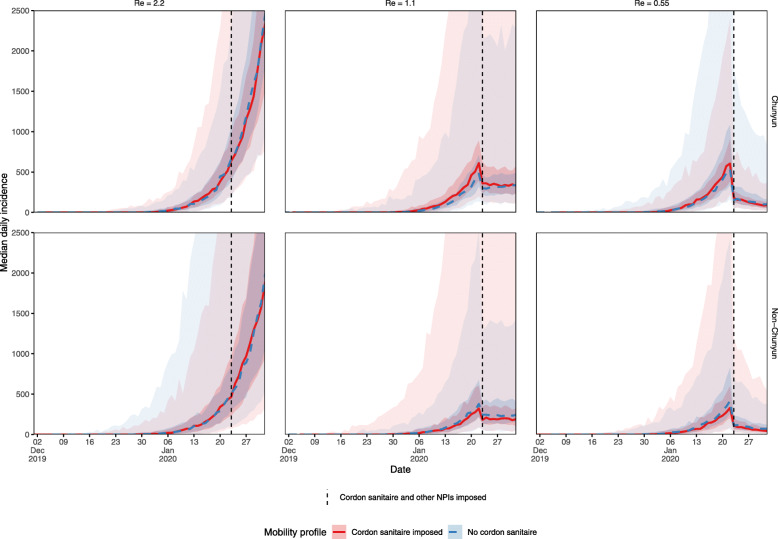
Median daily incidence of COVID-19 (shaded areas indicate 50% and 95% confidence intervals) in Beijing, for *Chunyun* vs. non-*Chunyun* and *cordon sanitaire* imposed (red, solid) vs. no *cordon sanitaire* (blue, dashed) and for varying values of the effective reproduction number *R*_*e*_, where *R*_*e*_ = 2.2 (no change, unmitigated local outbreak), reduced from 2.2 by 50% to 1.1 (mitigation of outbreak, *R*_*e*_ > 1), and 75% to 0.55 (suppression of outbreak, *R*_*e*_ < 1)

No substantial difference was observed in the daily incidence in the scenarios with and without travel restrictions in the four cities after the *cordon sanitaire* was imposed on 23 January; there were enough infected people to sustain local transmission in the absence of imported infections (Fig. 3 and Additional file 1: Figure S4). After the implementation of the *cordon sanitaire* on 23 January, the trajectory of the epidemic is determined primarily by reductions in *R*_*e*_ to simulate local transmission-reducing interventions. In an unmitigated outbreak where *R*_*e*_ remains at 2.2, incidence continues to increase exponentially in both scenarios; with *R*_*e*_ reduced to 1.1, incidence steadies; and with *R*_*e*_ reduced to 0.55, incidence decreased towards zero. The incidence after 23 January did not differ in scenarios with or without the implementation of the *cordon sanitaire*, and no additional effect was observed due to the *cordon sanitaire* after reducing *R*_*e*_.

No substantial differences were observed in the estimated cumulative number of infections by 1 February with and without *cordon sanitaire* in any of the four cities, after accounting for uncertainty resulting from the importation process and variability in the number of secondary cases resulting from an infected case (overdispersion) (Fig. 3).

**Fig. 4:**
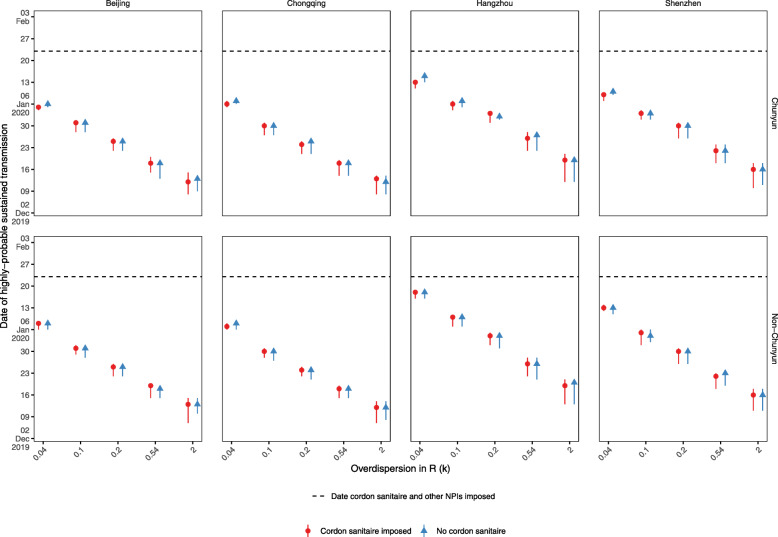
Estimated date on which the probability of an outbreak exceeds 95% in the 4 cities of interest, for *Chunyun* vs. non*-Chunyun* and *cordon sanitaire* imposed vs. no *cordon sanitaire* and for varying values of the overdispersion parameter *k* [15, 21, 22]. Median (and 95% CI) estimated cumulative number of infections on 1 March in the four cities of interest, *Chunyun* vs. non-*Chunyun*, *cordon sanitaire* imposed vs. no *cordon sanitaire*, and for varying values of *R*_*e*_, where *R* = 2.2 (no change, unmitigated local outbreak), reduced from 2.2 by 50% to 1.1 (mitigation of outbreak, *R* > 1), and 75% to 0.55 (suppression of outbreak, *R* < 1)

Decreasing the overdispersion parameter *k* from the baseline 0.1 to 0.04 [17] results in a delay to the likely date of an outbreak (Fig. 4); despite this, an outbreak was highly probable in all four cities prior to the date of the *cordon sanitaire*. Increasing *k* to 0.2 [17], 0.54 [16], and 2.0 (influenza-like) [21] further advanced the likely date of an outbreak.

## Discussion

By utilising publicly available mobility data to model the spread of the outbreak from Wuhan to other large population centres in China, we find that infected travellers from Wuhan likely led to local transmission in other major Chinese cities weeks before the *cordon sanitaire*. Cities with more travellers from Wuhan likely experienced higher incidence sooner. Modelling the trajectory of the outbreaks up to 1 February, in scenarios with and without the effect of the *cordon sanitaire*, we find no substantial differences in the cumulative number of infections generated.

By comparing *Chunyun* and non-*Chunyun* travel scenarios, no substantial difference was observed in terms of the cumulative number of infections generated by 1 February. This is likely due to the consistently high volume of travel these cities receive from Wuhan year round, resulting in enough infected travellers arriving to seed chains of transmission even during a period of regular travel volume. This however may differ in smaller cities which receive highly seasonal influxes of travellers from Wuhan relating to *Chunyun*.

The increase in mobility in 2020 compared to 2019 prior to LNY could be explained by a variety of factors, including year-to-year variations and potential factors related to COVID-19, such as the rumours of a rapidly growing outbreak and impending travel restrictions. In Northern Italy, a leaked COVID-19 plan might have driven thousands to flee south [25].

Our simulated number of arrivals of COVID-19 infections for Shenzhen by late January is broadly consistent with results shown in an observational study in Guangdong Province [26] (see Additional file 1: Supplementary Appendix 5, Figure S6 and Figure S7) [27–29]. However, the simulated number of locally transmitted cases around the same time is considerably higher than that observed. This disparity could be explained by testing programmes oversampling individuals with a recent travel history from highly affected areas, inflating the proportion of cases that were imported from outside Guangdong and missing cases which obtained the virus locally [30]. Furthermore, in scenarios when *R*_*e*_ was set to 0.55 after 23 January (Fig. 3 and Additional file 1: Figure S4), we also see case numbers decline at a similar rate and timeframe to the epidemic observed in Guangdong [23], suggesting stringent local NPIs played a key role in suppressing the outbreak.

In the formulation of the travel scenarios, we assumed that the Baidu Huiyan mobility index values were relative and linear and corresponded to 50,000 travellers per unit. This was based on widely quoted estimates of people leaving Wuhan and the inter-city capacity of the travel network [8, 11–14, 31, 32] (Additional file 1: Supplementary Appendix 1). However, the index may represent a different number of travellers, or the scale may even be non-linear and the result of a more complex function, but without other evidence we assume linearity, as have other studies [8, 13]. If we chose a higher scaling factor, similar to ones used in other studies [12, 13], it is likely that infected travellers would have arrived even earlier and in greater numbers to the four destination cities. Additionally, by reconstructing travel outflows for both dates outside of the observed range (22 November–31 December) and simulated aspects of our scenarios, i.e. *Chunyun* affected travel days in non-*Chunyun* scenarios, the actual travel pattern may not have been accurately represented. Further assumptions were also made surrounding the pairwise travel flows, as observed data was only available for 2020, and the travel flows between Wuhan and each other prefecture-level city may have differed in 2019. We only considered Wuhan to be the sole source of infected individuals, and we only accounted for travellers making single-leg journeys to their destination. As such, we may underestimate the number of infected persons arriving by not considering the number of travellers which may have stopped in an intermediate location, become infected, and then arrived at the destination to seed local transmission, or indeed infected travellers arriving from outside of Wuhan. Hence, most of our assumptions likely underestimated the number of travellers from Wuhan, and our conclusions would likely be the same even if the true number was higher. However, we also assumed that individuals would travel regardless of their infection status, which may overestimate the number of infections in destination cities.

In our model, we assume all chains of transmission are independent and populations in each city mix homogeneously. These assumptions are likely only valid in the early stages of an epidemic; however, as we only model the initial introduction of cases and their contact networks, the effect of changing these assumptions is unlikely to alter our conclusions. Moreover, the overdispersion parameter *k* likely captures the spread of *R*_*e*_ and acts to counter the assumed homogeneous population mixing. Reducing the overdispersion parameter *k* from 0.1 (~ 10% of individuals responsible for 80% of transmission [17]) to 0.04 (~ 5% of individuals responsible for 80% of transmission) resulted in a delay to the date of an outbreak, yet not past the date of the *cordon sanitaire*.

As recent studies have shown [8, 9, 33], strict physical distancing measures soon decreased the effective reproduction number to 1 or less in Wuhan and other cities in China. By incorporating this decrease into our model, we find that *cordon sanitaire* alone, implemented after outbreaks were likely to be established in other cities, was likely ineffective in stopping or slowing outbreaks of COVID-19 in other major population centres. To have a greater impact, the *cordon sanitaire* would need to be implemented earlier, as investigated in [34, 16], and be accompanied by other NPIs, such as general physical distancing and school and work closures [8, 23]. Similarly, it is unlikely that *cordon sanitaires* in other countries with well-established, geographically dispersed outbreaks will substantially delay COVID-19 spread. An open question is whether travel restrictions may be more efficacious to prevent or delay reintroductions after the lifting of other NPIs.

Whilst earlier restrictions on travel from Wuhan may have had a larger impact, in countries with a high-degree of inter-city travel, it may be difficult to implement such highly disruptive travel restrictions at an early stage of the epidemic, before local transmission has occurred in other cities. We find that local transmission in the four cities we studied (here defined as the probability of sustained transmission exceeding a 95% threshold) was most likely established between 1 January and 8 January; it was only on 8 January that the aetiology of the “mystery pneumonia” (which was not yet confirmed to spread from person-to-person [35]) was determined as a novel coronavirus, and the first death occurred [36]. It is difficult to see how the *cordon sanitaire* could have been justified any earlier, as almost every aspect of COVID-19 virology and epidemiology was unknown. Hence, it is likely that the sustained decline in COVID-19 incidence in other cities of China several months into the outbreak is primarily due to other public health measures to reduce the disease transmissibility, i.e. to reduce the reproduction number to 1 or below [9, 23, 33]. The *cordon sanitaire* may have been more efficacious in delaying outbreaks internationally, as the relative number of travellers is orders of magnitude lower [8, 37]; the same may also apply to lower-traffic destinations from Wuhan within China, such as small cities geographically distant from Wuhan, as observed in Tian et al. [8]. We found a mixed picture in these cities, where the *cordon sanitaire* may have been more efficacious at delaying or preventing outbreaks (Additional file 1: Figure S1 A-F). However, COVID-19 transmission dynamics may differ in comparison to large cities, and as such, we chose to focus on the effect of travel restrictions in large cities with large volumes of travel from Wuhan, where data on *R* and *k* from the early outbreak in Wuhan are likely generalisable. Furthermore, these destinations with low traffic from Wuhan are more likely to be seeded by outbreaks in other, comparatively closer, large cities first. Hence, our assumption of a single outbreak source would have been much less realistic.

Our estimated dates of introduction in other cities are earlier than those observed [1] and reported in other studies [38]. This is due in part to correction for underreporting, both by using the estimated daily prevalence in Wuhan from Kucharski et al. [9], which is significantly higher than the confirmed number of cases [20], and by not relying on reported cases in other provinces. The effect of underreporting is likely more pronounced early in the outbreak prior to a well-defined case definition or widespread testing [15]. Hence, reconstructing the early outbreak through a simulation approach was more appropriate in this setting.

We concur with Tian et al. 2020 [8] that prohibiting travel alone did not act to reduce the number of COVID-19 infections in four major cities outside of Wuhan or Hubei and that other local control measures were likely instrumental in reducing incidence. Likewise, Kraemer et al. [38] conclude that whilst a decrease in the growth rate was observed in large cities after the *cordon sanitaire* was imposed, this is difficult to disentangle from local control measures.

## Conclusion

In conclusion, the introduction of *cordon sanitaire*-type travel restrictions around a COVID-19 epidemic centre after community transmission is already occurring in other well-connected population centres on its own likely has little effect on altering their epidemic trajectories. Stringent NPIs in cities are more likely to have a bigger impact in reducing incidence and pressure on healthcare systems. Further research should examine the role of travel restrictions during the partial lifting of NPIs across China and elsewhere.

## Supplementary information

**Additional file 1 : Supplementary Appendix 1–5. Table S1** - Various scaling factors calculated from different sources. **Table S2** - Parameters used to estimate the total number of travellers leaving Wuhan and entering other prefecture-level cities for each scenario. **Table S3.** Bivariate regression results where y = number of imported cases into Guangdong (by date of symptom onset) and x = imported cases into Guangdong (by date of arrival), for an increasing amount of day lags. **Figure S1.** Date at which the mean probability of sustained transmission breaches 95% in each prefecture, by region (A-F). **Figure S2.** Location of Wuhan and the four cities of interest in mainland China. **Figure S3.** Delay distributions for the serial interval of COVID-19 infection from literature. **Figure S4.** Median daily incidence of COVID-19 in the four cities of interest. **Figure S5.** Median daily incidence of COVID-19 in the four cities of interest with alternative serial interval of mean 7.5 days (SD: 3.4). **Figure S6.** Estimated daily infected arrivals in Guangdong Province. **Figure S7.** Observed imported cases by date of symptom onset vs. predicted imported cases by date of arrival with a lag of 4 days.

## Data Availability

The mobility data was sourced from Baidu Haiyan migration dashboard at https://qianxi.baidu.com/. The code for this analysis is available on GitHub at https://github.com/bquilty25/wuhan_travel_restrictions.

## Abbreviations

*k*: Overdispersion parameter
LBS: Location-based service
LNY: Lunar New Year
NPIs: Non-pharmaceutical interventions
*R*_*e*_: Effective reproduction number
SI: Serial interval
UI: Uncertainty interval

## References

[1] World Health Organisation. Novel coronavirus (2019-nCoV) situation reports. https://www.who.int/emergencies/diseases/novel-coronavirus-2019/situation-reports. Accessed 30 Mar 2020.

[2] Virus-hit Chinese city shuts public transport. BBC News. 2020. https://www.bbc.com/news/world-asia-china-51215348. Accessed 1 Apr 2020.

[3] Il governo firma il decreto coronavirus: l’Italia divisa in 3 zone (Translated: The government signs the coronavirus decree: Italy divided into 3 areas). la Repubblica. 2020. https://www.repubblica.it/politica/2020/03/01/news/coronavirus_misure_governo-249980561/. Accessed 1 Apr 2020.

[4] Zurcher A. Trump’s virus travel ban on Europe comes into force. BBC News 2020. https://www.bbc.com/news/world-us-canada-51883728. Accessed 1 Apr 2020.

[5] Mateus AL, Otete HE, Beck CR, Dolan GP, Nguyen-Van-Tam JS. Effectiveness of travel restrictions in the rapid containment of human influenza: a systematic review. WHO. 2014. https://www.who.int/bulletin/volumes/92/12/14-135590/en/. Accessed 29 Jun 2020. 10.2471/BLT.14.135590 PMC4264390 25552771

[6] 襄阳火车站关闭, 湖北省最后一个地级市“封城”_媒体_澎湃新闻-The Paper. https://www.thepaper.cn/newsDetail_forward_5671283. Accessed 1 Apr 2020.

[7] Wang X, Liu C, Mao W, Hu Z, Gu L. Tracing the largest seasonal migration on Earth. arXiv. 2014. http://arxiv.org/abs/1411.0983. Accessed 20 Mar 2020.

[8] Tian H, Liu Y, Li Y, Wu C-H, Chen B, Kraemer MUG, et al. An investigation of transmission control measures during the first 50 days of the COVID-19 epidemic in China. Science. 2020. 10.1126/science.abb6105. 10.1126/science.abb6105 PMC7164389 32234804

[9] Kucharski AJ, Russell TW, Diamond C, Liu Y, Edmunds J, Funk S, et al. Early dynamics of transmission and control of COVID-19: a mathematical modelling study. Lancet Infect Dis 2020;0. doi:10.1016/S1473-3099(20)30144-4. 10.1016/S1473-3099(20)30144-4 PMC7158569 32171059

[10] Baidu. 首页-百度地图慧眼 (Translated: Baidu map). https://huiyan.baidu.com/. Accessed 1 Apr 2020.

[11] Sanche S, Lin YT, Xu C, Romero-Severson E, Hengartner N, Ke R. The novel coronavirus, 2019-nCoV, is highly contagious and more infectious than initially estimated. medRxiv. 2020. doi:10.1101/2020.02.07.20021154.

[12] Cao Z, Zhang Q, Lu X, Pfeiffer D, Wang L, Song H, et al. Incorporating Human Movement Data to Improve Epidemiological Estimates for 2019-nCoV. medRxiv. 2020. 10.1101/2020.02.07.20021071.

[13] Zhou C. Evaluating new evidence in the early dynamics of the novel coronavirus COVID-19 outbreak in Wuhan, China with real time domestic traffic and potential asymptomatic transmissions. medRxiv. 2020. 10.1101/2020.02.15.20023440.

[14] 春运前十天, 武汉铁公空发送400余万人次, 公共交通运送8000余万人次_首页武汉_新闻中心_长江网 (Translated: Ten days before the Spring Festival, Wuhan Railway Express sent more than 4 million passengers, and public transportation delivered more than 80 million passengers.). http://news.cjn.cn/sywh/202001/t3539167.htm. Accessed 23 Mar 2020.

[15] Using a delay-adjusted case fatality ratio to estimate under-reporting. CMMID Repository. 2020. https://cmmid.github.io/topics/covid19/severity/global_cfr_estimates.html. Accessed 25 Mar 2020.

[16] Riou J, Althaus CL (2020). Pattern of early human-to-human transmission of Wuhan 2019 novel coronavirus (2019-nCoV), December 2019 to January 2020. Eurosurveillance..

[17] Endo A, Centre for the Mathematical Modelling of Infectious Diseases COVID-19 Working Group, Abbott S, Kucharski AJ, Funk S. Estimating the overdispersion in COVID-19 transmission using outbreak sizes outside China. Wellcome Open Res. 2020;5:67. 10.12688/wellcomeopenres.15842.1 PMC7338915 32685698

[18] Nishiura H, Linton NM, Akhmetzhanov AR (2020). Serial interval of novel coronavirus (COVID-19) infections. Int J Infect Dis.

[19] Li Q, Guan X, Wu P, Wang X, Zhou L, Tong Y, et al. Early transmission dynamics in Wuhan, China, of novel coronavirus–infected pneumonia. N Engl J Med. 2020;382:1199–207. 10.1056/NEJMoa2001316 PMC7121484 31995857

[20] Hartfield M, Alizon S (2013). Introducing the outbreak threshold in epidemiology. PLoS Pathog.

[21] Lloyd-Smith JO, Schreiber SJ, Kopp PE, Getz WM (2005). Superspreading and the effect of individual variation on disease emergence. Nature..

[22] Jombart T, Cori A, Kamvar ZN, Schumacher D. epitrix: Small helpers and tricks for epidemics analysis. 2019. https://CRAN.R-project.org/package=epitrix. Accessed 29 May 2020.

[23] Prem K, Liu Y, Russell TW, Kucharski AJ, Eggo RM, Davies N, et al. The effect of control strategies to reduce social mixing on outcomes of the COVID-19 epidemic in Wuhan, China: a modelling study. Lancet Public Health 2020;0. doi:10.1016/S2468-2667(20)30073-6. 10.1016/S2468-2667(20)30073-6 PMC7158905 32220655

[24] Project Future Case Incidence. http://www.repidemicsconsortium.org/projections/. Accessed 11 Mar 2020.

[25] Leaked coronavirus plan to quarantine 16m sparks chaos in Italy. The Guardian. 2020. https://www.theguardian.com/world/2020/mar/08/leaked-coronavirus-plan-to-quarantine-16m-sparks-chaos-in-italy. Accessed 27 Mar 2020.

[26] Lu J, du Plessis L, Liu Z, Hill V, Kang M, Lin H, et al. Genomic epidemiology of SARS-CoV-2 in Guangdong Province, China. Cell. 2020;181:997–1003.e9. 10.1016/j.cell.2020.04.023 PMC7192124 32359424

[27] Backer JA, Klinkenberg D, Wallinga J. Incubation period of 2019 novel coronavirus (2019-nCoV) infections among travellers from Wuhan, China, 20–28 January 2020. Eurosurveillance. 2020;25. 10.2807/1560-7917.ES.2020.25.5.2000062. 10.2807/1560-7917.ES.2020.25.5.2000062 PMC7014672 32046819

[28] Wölfel R, Corman VM, Guggemos W, Seilmaier M, Zange S, Müller MA (2020). Virological assessment of hospitalized patients with COVID-2019. Nature..

[29] Chau NVV, Thanh Lam V, Thanh Dung N, Yen LM, Minh NNQ, Hung LM, et al. The natural history and transmission potential of asymptomatic SARS-CoV-2 infection. Clin Infect Dis. 10.1093/cid/ciaa711. 10.1093/cid/ciaa711 PMC7314145 32497212

[30] China CDC. 新型冠状病毒肺炎流行病学调查指南 (Translated: Guidelines for the epidemiological investigation of new coronavirus pneumonia). http://www.chinacdc.cn/jkzt/crb/zl/szkb_11803/jszl_11815/202003/W020200309540843000869.pdf. Accessed 27 Mar 2020.

[31] 北京三大火车站迎节前客流最高峰 预计62万人次乘火车离京-新华网 (Translated: Beijing’s three major railway stations have the highest peak passenger flow before the festival, and 620,000 people are expected to leave Beijing by train). http://www.xinhuanet.com/fortune/2019-02/02/c_1124077797.htm. Accessed 14 Apr 2020.

[32] 春运期间首都机场预计进出港旅客1151万人次--财经-- (Translated: During the Spring Festival transport, Capital Airport is expected to have 11.51 million passengers in and out of Hong Kong). http://finance.people.com.cn/n1/2019/0121/c1004-30581248.html. Accessed 14 Apr 2020.

[33] Zhang J, Litvinova M, Liang Y, Wang Y, Wang W, Zhao S (2020). Age profile of susceptibility, mixing, and social distancing shape the dynamics of the novel coronavirus disease 2019 outbreak in China.

[34] Lai S, Ruktanonchai NW, Zhou L, Prosper O, Luo W, Floyd JR, et al. Effect of non-pharmaceutical interventions for containing the COVID-19 outbreak in China. medRxiv. 2020. doi:10.1101/2020.03.03.20029843.

[35] Chan JF-W, Yuan S, Kok K-H, To KK-W, Chu H, Yang J, et al. A familial cluster of pneumonia associated with the 2019 novel coronavirus indicating person-to-person transmission: a study of a family cluster. Lancet 2020;395:514–523. 10.1016/S0140-6736(20)30154-9 PMC7159286 31986261

[36] Qin A, Hernández JC. China reports first death from new virus. The New York Times. 2020. https://www.nytimes.com/2020/01/10/world/asia/china-virus-wuhan-death.html. Accessed 26 Mar 2020.

[37] Chinazzi M, Davis JT, Ajelli M, Gioannini C, Litvinova M, Merler S, et al. The effect of travel restrictions on the spread of the 2019 novel coronavirus (COVID-19) outbreak. Science. 2020. 10.1126/science.aba9757. 10.1126/science.aba9757 PMC7164386 32144116

[38] Kraemer MUG, Yang C-H, Gutierrez B, Wu C-H, Klein B, Pigott DM, et al. The effect of human mobility and control measures on the COVID-19 epidemic in China. Science. 2020. 10.1126/science.abb4218. 10.1126/science.abb4218 PMC7146642 32213647

