# Supplementary material for: The effect of travel restrictions on the geographical spread of COVID-19 between large cities in China: a modelling study
Source: BMC Med. 2020 Aug 19;18:259. doi: 10.1186/s12916-020-01712-9 (PMC7437104; doi:10.1186/s12916-020-01712-9)
Supplement: Supplementary file 1 — Additional file 1 : Supplementary Appendix 1–5. Table S1 - Various scaling factors calculated from different sources. Table S2 - Parameters used to estimate the total number of travellers leaving Wuhan and entering other prefecture-level cities for each scenario. Table S3. Bivariate regression results where y = number of imported cases into Guangdong (by date of symptom onset) and x = imported cases into Guangdong (by date of arrival), for an increasing amount of day lags. Figure S1. Date at which the mean probability of sustained transmission breaches 95% in each prefecture, by region (A-F). Figure S2. Location of Wuhan and the four cities of interest in mainland China. Figure S3. Delay distributions for the serial interval of COVID-19 infection from literature. Figure S4. Median daily incidence of COVID-19 in the four cities of interest. Figure S5. Median daily incidence of COVID-19 in the four cities of interest with alternative serial interval of mean 7.5 days (SD: 3.4). Figure S6. Estimated daily infected arrivals in Guangdong Province. Figure S7. Observed imported cases by date of symptom onset vs. predicted imported cases by date of arrival with a lag of 4 days. [file 12916_2020_1712_MOESM1_ESM.docx]

## Supplementary Appendix

#### 1. Estimating the scaling factor

To estimate the absolute number of daily travellers leaving Wuhan from Baidu’s migration index, we needed a suitable scaling factor to convert the index score to the absolute number of travellers. In lieu of other evidence, we assumed this relationship to be linear cohering with other studies [[8, 13]](https://www.zotero.org/google-docs/?7ihFzJ). We synthesised estimates from a number of sources (Table S1) in order to select the most viable result. In each case the scaling factor was calculated using the following equation:

[
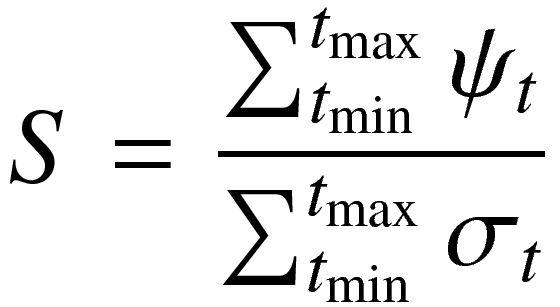
](http://www.sciweavers.org/tex2img.php?bc=White&fc=Black&im=jpg&fs=78&ff=txfonts&edit=0&eq=%20S%20%3D%20%5Cfrac%7B%5Csum_%7Bt_%7B%5Ctext%7Bmin%7D%7D%7D%5E%7Bt_%7B%5Ctext%7Bmax%7D%7D%7D%5Cpsi_%7Bt%7D%20%7D%7B%5Csum_%7Bt_%7B%5Ctext%7Bmin%7D%7D%7D%5E%7Bt_%7B%5Ctext%7Bmax%7D%7D%7D%5Csigma_%7Bt%7D%20%7D%20#0)

Where the sum of the daily estimated number of travellers *ψ_t,_*leaving Wuhan for the dates *t_min_* to *t_max_*, divided by the sum of the daily outflow index from Wuhan σ_t_, for the same date range, equals the scaling factor *S*.

*Table S1 - Various scaling factors calculated from different sources.*

| **Reference** | **Date range (*t_min_* to *t_max_*)** | **Sum of traveller numbers leaving Wuhan (*ψ_t_*)** | **Sum of Baidu travel index leaving Wuhan (*σ_t_*)** | **Estimated scaling factor (*S*)** |
| --- | --- | --- | --- | --- |
| Tian (2020) [[8]](https://www.zotero.org/google-docs/?1ner3R) | Jan 11 - Jan 25 | 4,325,563* | 105.69 | 40,926.89 |
| Sanche (2020) [[11]](https://www.zotero.org/google-docs/?gbBWMw) | Jan 10 - Jan 23 | 5,000,000 | 107.12 | 46,676.62 |
| News report (2020) [[14]](https://www.zotero.org/google-docs/?6JbSJA) | Jan 10 - Jan 20 | 4,098,600 | 73.40 | 55,839.24 |
| Cao (2020) [[12]](https://www.zotero.org/google-docs/?JIY0LQ) | Jan 16 - Jan 22 | 7,014,199* | 58.45 | 120,003.40 |
| Zhou (2020) [[13]](https://www.zotero.org/google-docs/?Phz5ZT) | Unknown | Unknown | Unknown | 138,412.00 |

* Based on data extracted from figures, subject to slight error.

Combining evidence from the first three sources in Table S1, we chose a scaling factor of 50,000. This assumes each unit of Baidu’s migration index corresponds to 50,000 outbound travellers. This produced the most reasonable outbound travel volume estimates, using scaling factors of 120,003.40 and 138,412, found in Cao (2020) and Zhou (2020) respectively, yielded unrealistically large travel magnitudes. These scaling factors would suggest that on Beijing’s single busiest day of *Chunyun* (23 Jan, 2020), in excess of 2.8 million people left the city. This is substantially larger than Beijing's maximum daily outbound travel capacity by air and rail, which is estimated to be 920,000 daily passengers [[31, 32]](https://www.zotero.org/google-docs/?bIp2GH). This estimate does not consider passengers traveling by road, however this form of transportation accounts for a relatively small proportion of the total inter-prefecture travel.

#### 2. Estimating number of infected travellers

The number of travellers arriving in each city from Wuhan is summarised as:

[
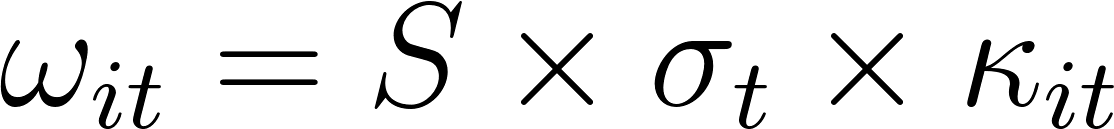
](https://www.codecogs.com/eqnedit.php?latex=%20%5Comega_%7Bit%7D%20%3D%20S%20%5Ctimes%20%5Csigma_%7Bt%7D%20%5Ctimes%20%5Ckappa_%7Bit%7D%20#0)

Where *S* is the scaling factor, *σ_t_* is the total daily outflow index from Wuhan, *κ_it_* is the daily proportion of outflow entering each city *i*, and *ω_it_* is the daily number of total arrivals from Wuhan in city *i*.

The number of daily infected arrivals to a given prefecture *i* is simulated by making 100 draws from a Poisson process:

[
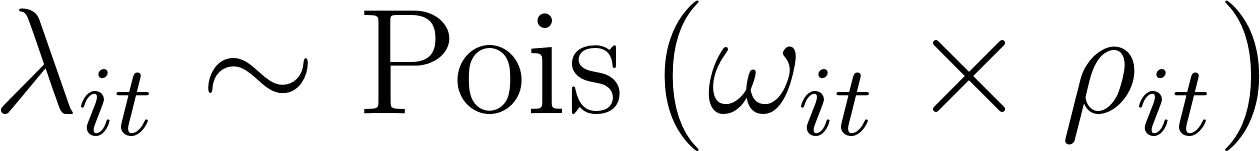
](https://www.codecogs.com/eqnedit.php?latex=%5Clambda_%7Bit%7D%20%5Csim%20%5Ctext%7BPois%7D%5Cleft(%20%5Comega_%7Bit%7D%5Ctimes%20%5Crho_%7Bit%7D%20%5Cright)%250)

Where *ω_it_* is the daily estimated travel from Wuhan to prefecture *i* on day *t, ρ_it_* is the daily prevalence in Wuhan, and *λ_it_* is the number of infected individuals arriving per day.

#### 3. Travel flow scenario formulation

The observed travel outflow from Wuhan in 2019 and 2020 were matched by the date of the Lunar New Year in 2020 so as to align the *Chunyun* travel patterns. Each scenario is driven by differences in the parameters used to estimate the total daily number of travellers arriving from Wuhan in a given prefecture-level city. In all scenarios the scaling factor was assumed to be constant at 50,000. Differences between scenarios are summarised in the table and equations below:

*Table S2 - Parameters used to estimate the total number of travellers leaving Wuhan and entering other prefecture-level cities for each scenario.*

| **Scenario and description** | **Daily outflow from Wuhan (σ_t_)** | **Daily proportion of travellers leaving Wuhan and entering each prefecture-level city (κ_it_)** |
| --- | --- | --- |
| **Scenario 1** - *Chunyun* & *cordon sanitaire* | 22 Nov - 31 Dec: [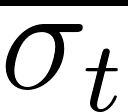](https://www.codecogs.com/eqnedit.php?latex=%5Coverline%7B%5Csigma_%7Bt%7D%7D#0) *  1 Jan - 1 Mar: **σ_t_** (Observed 2020) | 22 Nov - 31 Dec: [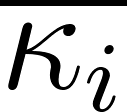](https://www.codecogs.com/eqnedit.php?latex=%5Coverline%7B%5Ckappa%20_%7Bi%7D%7D%20#0)^†^  1 Jan - 1 Mar: **κ_it_**  (Observed 2020) |
| **Scenario 2** - *Chunyun* & no *cordon sanitaire* | 22 Nov - 31 Dec: [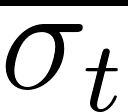](https://www.codecogs.com/eqnedit.php?latex=%5Coverline%7B%5Csigma_%7Bt%7D%7D#0) *  1 Jan - 1 Mar: **σ_t_** (Observed 2019^^^) | 22 Nov - 31 Dec: [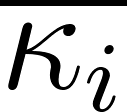](https://www.codecogs.com/eqnedit.php?latex=%5Coverline%7B%5Ckappa%20_%7Bi%7D%7D%20#0)^†^  1 Jan - 19 Jan: **κ_it_**  (Observed 2020)  20 Jan - 1 Mar: [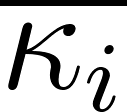](https://www.codecogs.com/eqnedit.php?latex=%5Coverline%7B%5Ckappa%20_%7Bi%7D%7D%20#0)^†^ |
| **Scenario 3** - No *Chunyun* & *cordon sanitaire* | 22 Nov - 5 Jan: [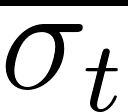](https://www.codecogs.com/eqnedit.php?latex=%5Coverline%7B%5Csigma_%7Bt%7D%7D#0) *  6 Jan - 10 Jan: **σ_t_**  (Observed 2019^^^)  11 Jan - 23 Jan: [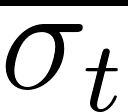](https://www.codecogs.com/eqnedit.php?latex=%5Coverline%7B%5Csigma_%7Bt%7D%7D#0) *  24 Jan - 1 Mar: **σ_t_**  (Observed 2020) | 22 Nov - 23 Jan: [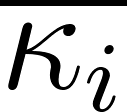](https://www.codecogs.com/eqnedit.php?latex=%5Coverline%7B%5Ckappa%20_%7Bi%7D%7D%20#0)^†^  24 Jan - 1 Mar: **κ_it_**  (Observed 2020) |
| **Scenario 4** - No *Chunyun* & no *cordon sanitaire* | 22 Nov - 5 Jan: [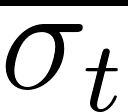](https://www.codecogs.com/eqnedit.php?latex=%5Coverline%7B%5Csigma_%7Bt%7D%7D#0) *  6 Jan - 10 Jan: **σ_t_**  (Observed 2019^^^)  11 Jan - 7 Feb: [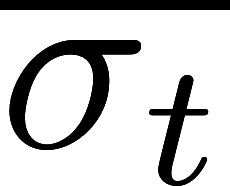](http://www.texrendr.com/?eqn=%5Coverline%7B%5Csigma_%7Bt%7D%7D#0) *  Feb 8 - 1 Mar: **σ_t_**  (Observed 2019^^^) | 22 Nov - 1 Mar: [**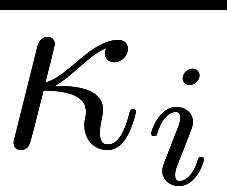**](http://www.texrendr.com/?eqn=%5Coverline%7B%5Ckappa%20_%7Bi%7D%7D%20#0)^†^ |

^^^ Equivalent *Chunyun* dates aligned to the 2020 calendar.

* See equation 1.

^†^ See equation 2.

Equation 1:

[
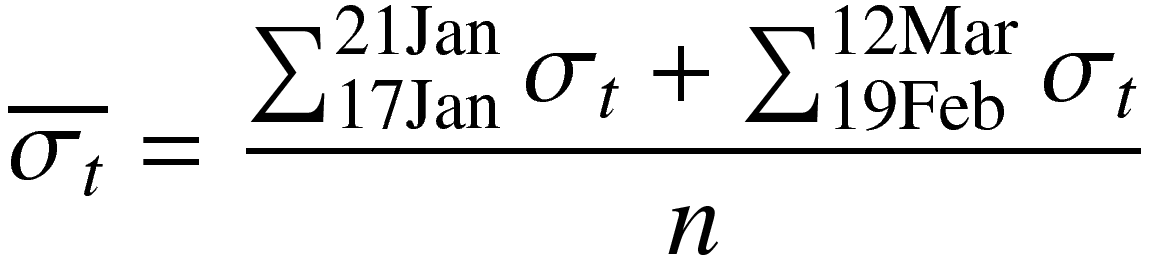
](http://www.sciweavers.org/tex2img.php?bc=White&fc=Black&im=jpg&fs=78&ff=txfonts&edit=0&eq=%20%5Coverline%7B%5Csigma_%7Bt%7D%7D%20%3D%20%5Cfrac%7B%5Csum_%7B17%20%5Ctext%7BJan%7D%7D%5E%7B21%20%5Ctext%7BJan%7D%7D%20%5Csigma%20_%7Bt%7D%2B%20%5Csum_%7B19%20%5Ctext%7BFeb%7D%7D%5E%7B12%20%5Ctext%7BMar%7D%7D%5Csigma%20_%7Bt%7D%7D%7Bn%7D%20#0)

The above equation estimates the mean daily outflow index from Wuhan in 2019 for the following dates; 17 Jan - 21 Jan and 19 Feb - 12 Mar. These dates are understood to be days of regular travel volume, and as such can be used to construct an estimate of an average travel flow for a representative non-*Chunyun* period.

Equation 2:

[
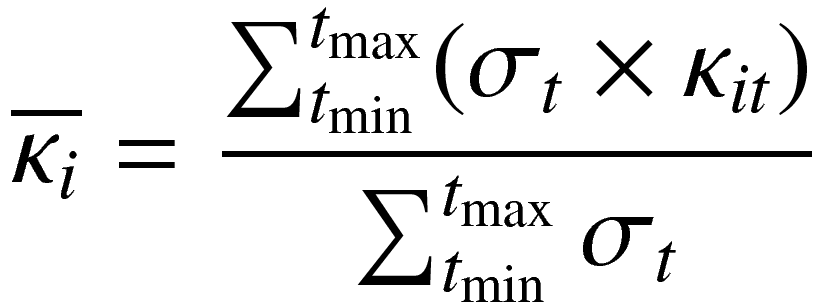
](http://www.sciweavers.org/tex2img.php?bc=White&fc=Black&im=jpg&fs=78&ff=txfonts&edit=0&eq=%20%5Coverline%7B%5Ckappa%20_%7Bi%7D%7D%20%3D%20%5Cfrac%7B%5Csum_%7Bt_%7B%5Ctext%7Bmin%7D%7D%7D%5E%7Bt_%7B%5Ctext%7Bmax%7D%7D%7D(%5Csigma%20_%7Bt%7D%5Ctimes%20%5Ckappa%20_%7Bit%7D)%7D%7B%5Csum_%7Bt_%7B%5Ctext%7Bmin%7D%7D%7D%5E%7Bt_%7B%5Ctext%7Bmax%7D%7D%7D%5Csigma%20_%7Bt%7D%7D%20#0)

Equation 2 approximates the general daily proportion of travellers leaving Wuhan and entering a given city *i*. For the length of the study period (*t_min_* to *t_max_*), we take the sum of the estimated travel flow leaving Wuhan and entering city *i* (*σ_t_ ✕ κ_it_*) and divide it by the sum of the total outflow from Wuhan *σ_t_* over the same period. This was a key assumption as the pairwise travel flows between Wuhan and each other prefecture-level city was only available between 1 Jan - 1 Mar, 2020. Therefore this approximation of general flow magnitude was used for both out of date ranges (22 Nov - 31 Dec) and simulated aspects of our scenarios i.e. *Chunyun* affected travel days in non-*Chunyun* scenarios.

#### 4. Probability of sustained transmission (outbreak threshold)

The probability of sustained transmission was calculated using methods detailed in the Supplement of Hartfield and Alizon 2013 [[20]](https://www.zotero.org/google-docs/?ucrMEY), which we will briefly summarise here.

Given a secondary case distribution with mean *R* and dispersion parameter *k*, the individual probability of a outbreak *q* can be numerically solved by:

[
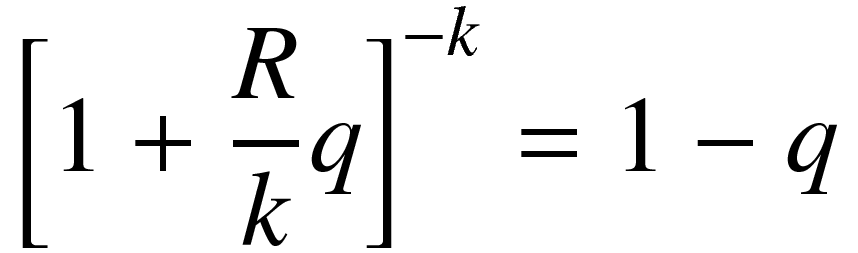
](http://www.sciweavers.org/tex2img.php?bc=White&fc=Black&im=jpg&fs=78&ff=txfonts&edit=0&eq=%20%5Cleft%5B1%2B%5Cfrac%7BR%7D%7Bk%7Dq%5Cright%5D%5E%7B-k%7D%20%3D%201-q%20#0)

The number of individuals required (*i*) such that the probability at least one of them causes an outbreak is then:

[
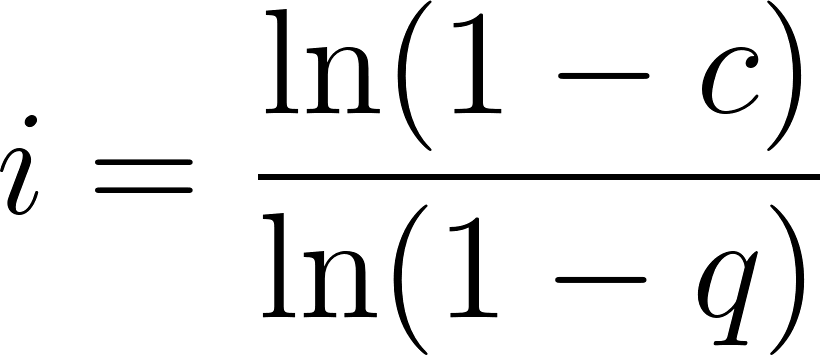
](https://www.codecogs.com/eqnedit.php?latex=i%20%3D%20%5Cfrac%7B%5Cln(1-c)%7D%7B%5Cln(1-q)%7D#0)

Where *c* (the outbreak threshold) was chosen as 0.95.

The impact of travel restrictions was assessed by comparing the daily probability of an outbreak occurring *O* for 2020 (restrictions imposed) and 2019 (“business-as-usual”).

#### 5. Comparison with Observational Study in Guangdong

Whilst the modelling presented in this study can be a useful tool to help understand the dynamics and spread of COVID-19 between large cities in China, it is important to contextualise the results against those observed and reported in other studies. To do this, we compared our predicted number of daily imported cases (by date of arrival) against the number of daily reported imported cases (by date of symptom onset) reported by Lu et al. (2020) in Guangdong from late December 2019 to early February 2020 (Figure S6). The two time-series show striking similarities by both rising and falling at broadly comparable rates, albeit with different magnitudes, and a delay of approximately 4 days. After adjusting for a 4 day lag, when statistically compared in a bivariate linear regression, results yield an adjusted *R^2^* value of 0.67 signifying that our estimated number of imported cases, by date of arrival has a reasonably strong ability to explain variance in the reported case numbers by date of symptom onset (Figure S7). This relationship can be summarised by the following formula:

*Observed imported cases = 7.83883 + (predicted arrival cases lag_4 * 0.58174)*

This can be interpreted as approximately 58.2% of the total predicted arrivals plus 7.84 cases, are able to explain 67% of the variation in the observed reported case numbers, by date of symptom onset. We further examined different values of lag to see if this would improve the models fit. Full results of this can be seen in Table S3. In terms of maximising the adjusted *R^2^* value, a lag of four days appears optimum. A lag of zero days produces a substantially worse fit, suggesting as expected that there is a delay before an infected arrival gets reported. Although this explains a large proportion of the variation in the observed cases numbers by symptom onset, a substantial part remains unknown and is potentially attributable to several key differences in the definition of our predicted case numbers and the observed reported values.

Table S3 - Bivariate regression results where y = number of imported cases into Guangdong (by date of symptom onset) and x = imported cases into Guangdong (by date of arrival), for an increasing amount of day lags.

| **Independent variable (x)** | **Intercept** | **Effect size** | **Adjusted R^2^** |
| --- | --- | --- | --- |
| Imported cases by date of arrival | 14.2174 | 0.3438 | 0.20 |
| Imported cases by date of arrival, lag 1 day | 12.06987 | 0.41871 | 0.32 |
| Imported cases by date of arrival, lag 2 day | 10.05409 | 0.49544 | 0.47 |
| Imported cases by date of arrival, lag 3 day | 8.61880 | 0.55539 | 0.60 |
| Imported cases by date of arrival, lag 4 day | 7.83993 | 0.58174 | 0.67 |
| Imported cases by date of arrival, lag 5 day | 8.76404 | 0.56087 | 0.63 |
| Imported cases by date of arrival, lag 6 day | 10.67585 | 0.50511 | 0.52 |

Firstly, the case data presented in Lu et al. (2020) is defined as somebody “with travel history from Hubei or other epidemic regions and did not have close contact with local positive cases in the 14 days preceding illness onset”. At the beginning of this pandemic, we can assume most domestically imported cases in Guangdong are from Hubei and not other provinces. However, in our study we explicitly only model cases arriving from Wuhan, one of 17 prefecture-level units in the Hubei province. Wuhan is the only location with prevalence estimates during that time, and travel was completely stopped on Jan 23rd. Travellers from other prefectures in Hubei were able to travel out until Jan 26-27th, based on our knowledge of local movement restriction policies. Thus, travellers in our study (compared to those in Lu et al. (2020)) not only came from different locations, but also travelled at different times. Since the proportion of reported cases imported from Wuhan were not presented in Lu et al. (2020), comparisons between the observed reported importations and our predicted importations would potentially not capture the whole story.

Secondly, our outcome is generated in terms of the date of arrival into a given city. In order to convert this date to a symptom onset date (which is only available for symptomatic individuals) and make it more comparable to Lu et al. 2020, we need to consider the following:

1. Each individuals’ incubation period (time from infection to symptoms onset), estimated to have 95% range of 2.1 to 11.1 days, with a median of 6.4 [[27]](https://www.zotero.org/google-docs/?7Jrz0t). For simplicity, we will use the median (t1)
2. Each individuals’ recovery time (time from symptoms onset to loss of infectious period). This may last to around 10 days, with a median 7-8 days [[28]](https://www.zotero.org/google-docs/?jDZdTN). For simplicity, we will use 8 days (t2).
3. Cases are confirmed based on being PCR positive, and not based on their infectiousness. Cases could be PCR positive for up to three weeks after the time of infection [[28]](https://www.zotero.org/google-docs/?w8zcGY). For simplicity, we will use 14 days (t3).
4. At the beginning of the outbreak, syndromic surveillance was not fully utilised, so symptomatic individuals may still have travelled. This means, for a symptomatic individual arriving on a given date d, the onset date could be anywhere from d-t2 or d-t3 to d+t1. This is roughly a 2-3 week range (t1+t2 or t1+t3). The entire time horizon we focus on is roughly 6 weeks. So the uncertainty is quite substantial, even when we only consider the point estimates in 1-3.
5. Probability of detection, which varies by travel time [[29]](https://www.zotero.org/google-docs/?yOXDpP). For instance, travellers that arrive in Guangdong more than a week after symptom onset are less likely to be detected than those arriving in Guangdong 1 day after symptom onset although, at the time, they are equally likely to travel.

Based on these factors, we believe that the direct comparison between imported infected travellers by their arrival date, and imported cases by their symptom onset date should be interpreted with extreme caution, even after accounting for lag. A more accurate comparison could be done using a mechanistic model explicitly accounting for the relevant disease parameters presented above (and their associated uncertainties), and with more accurate locations of traveller origin.

#### 6. Supplementary figures and tables

*
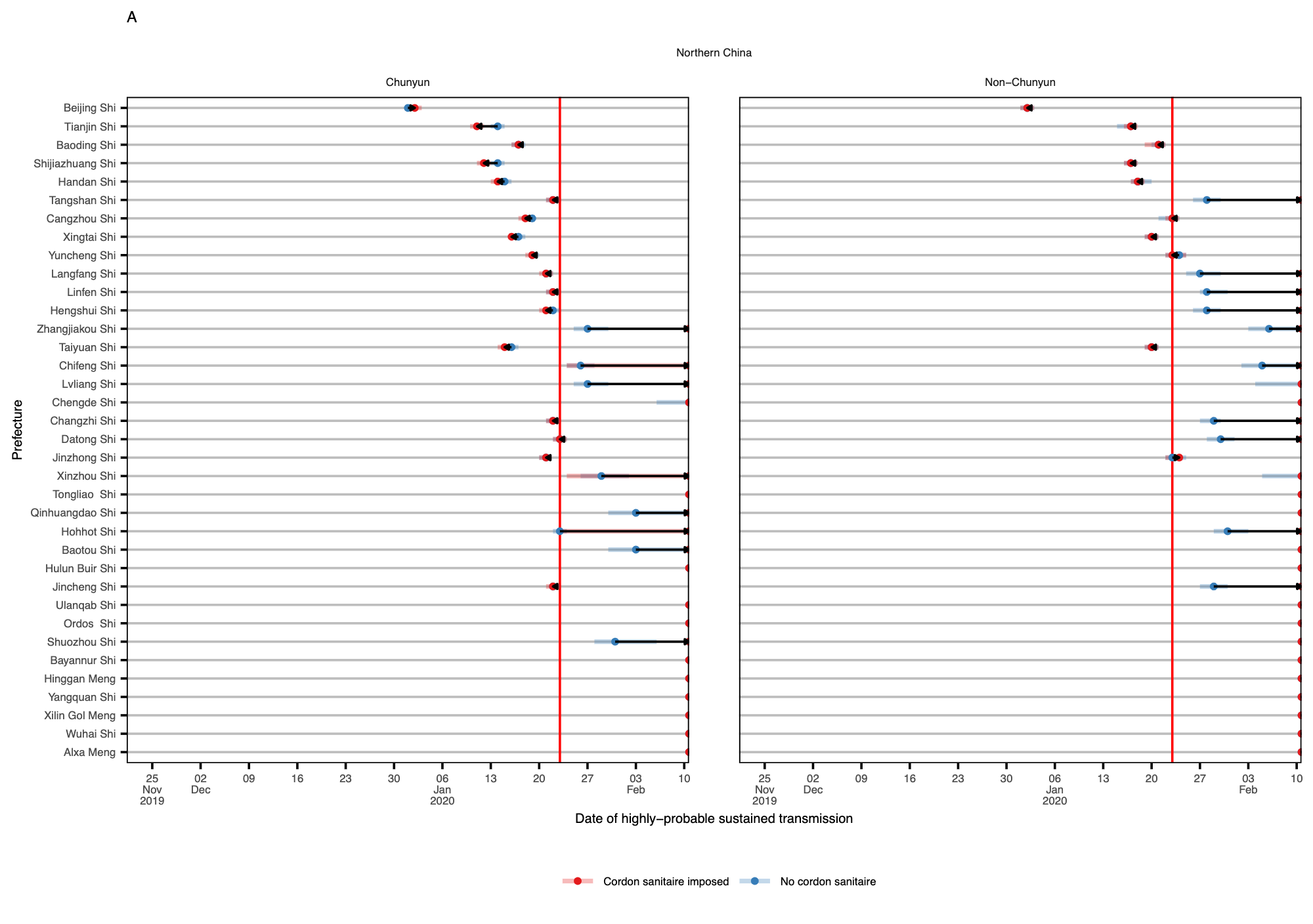
*

*
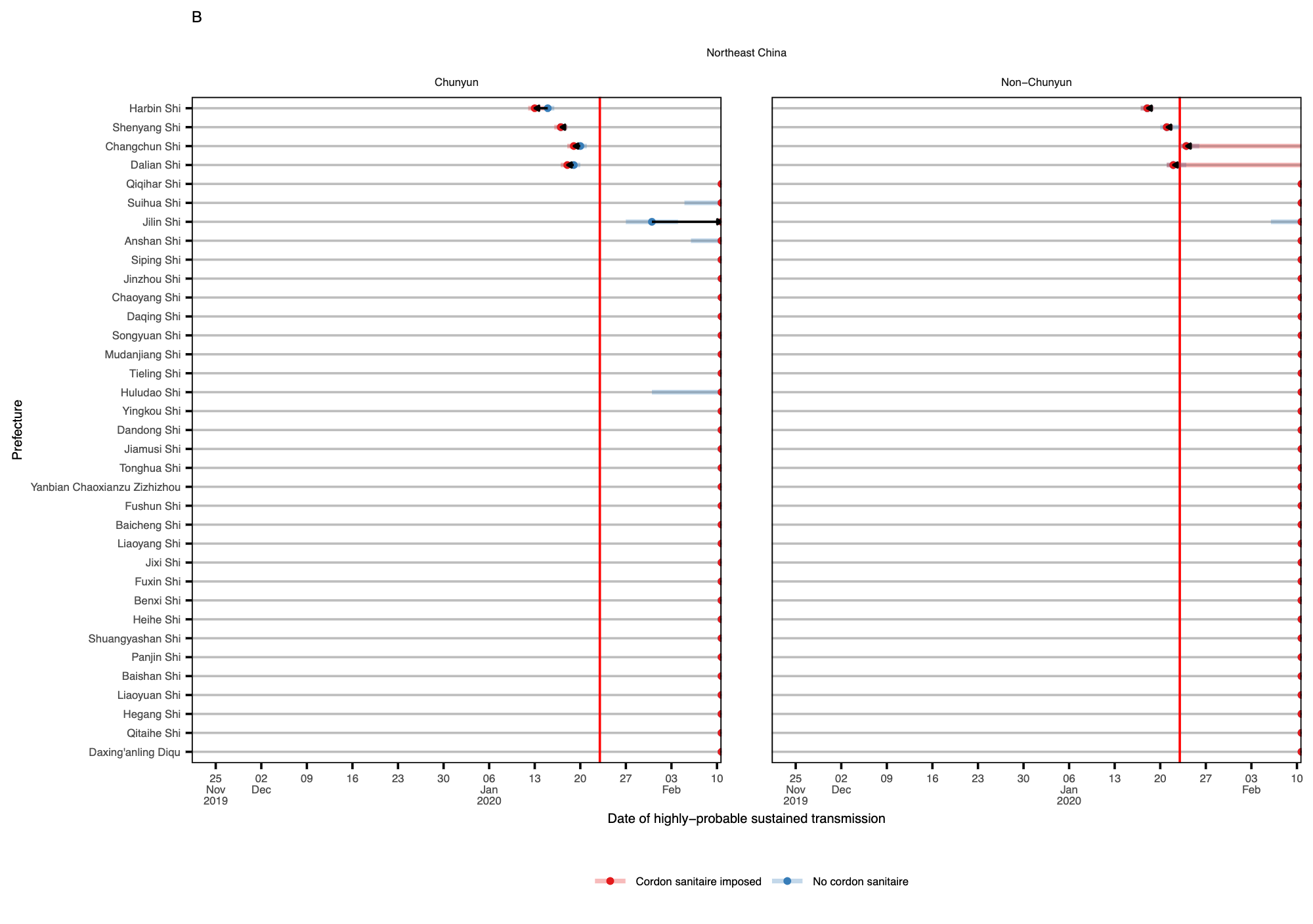
*

*
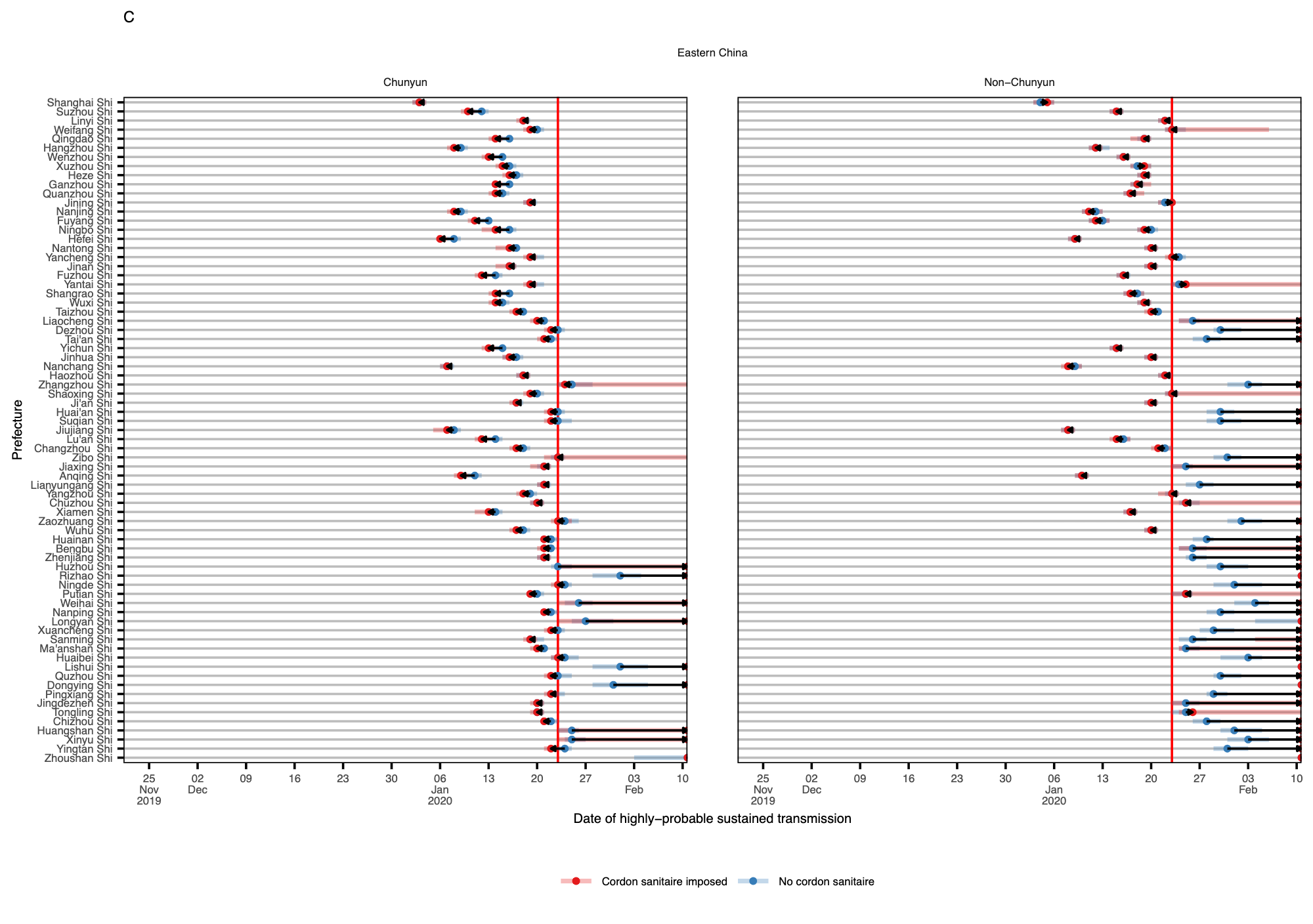

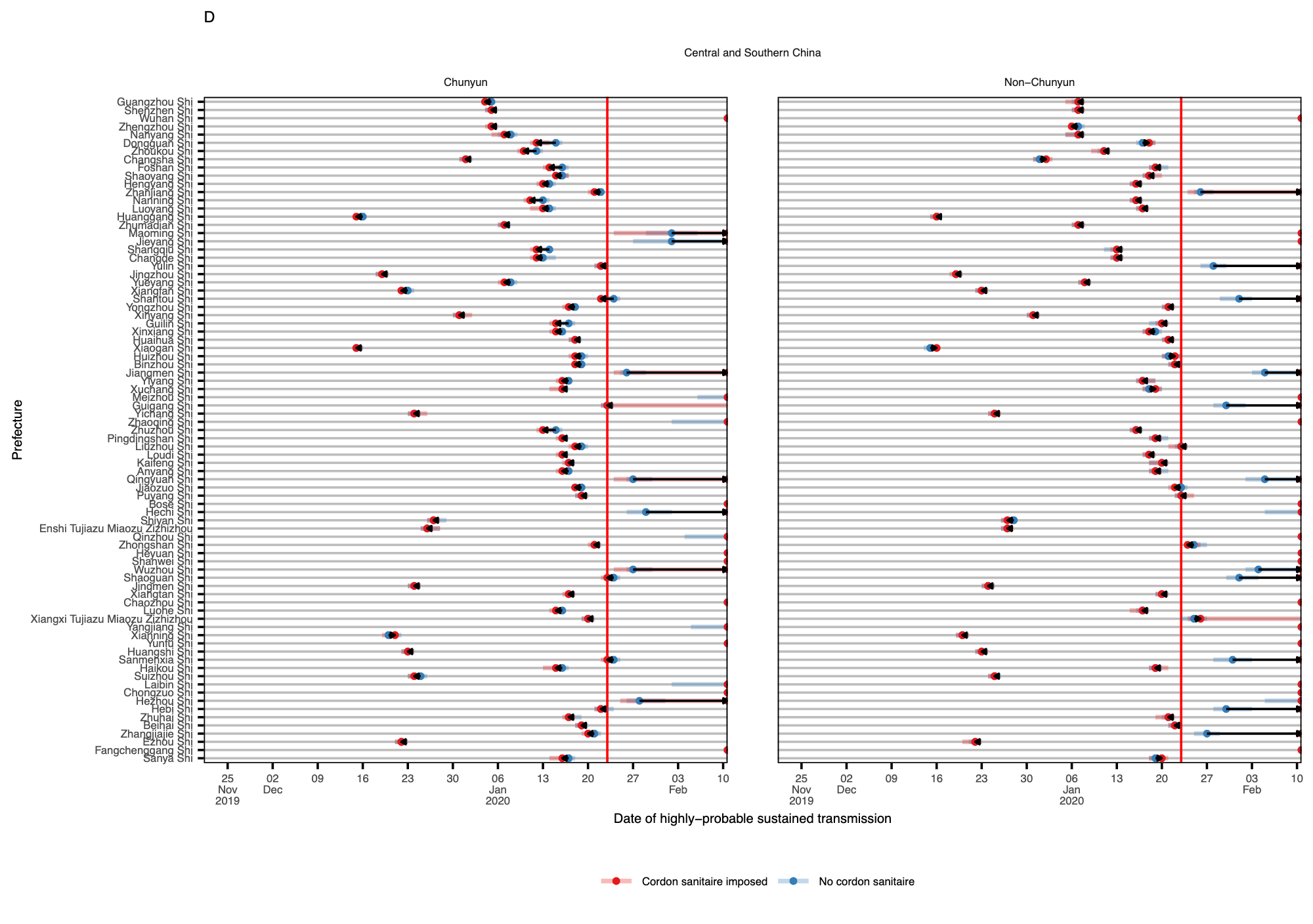

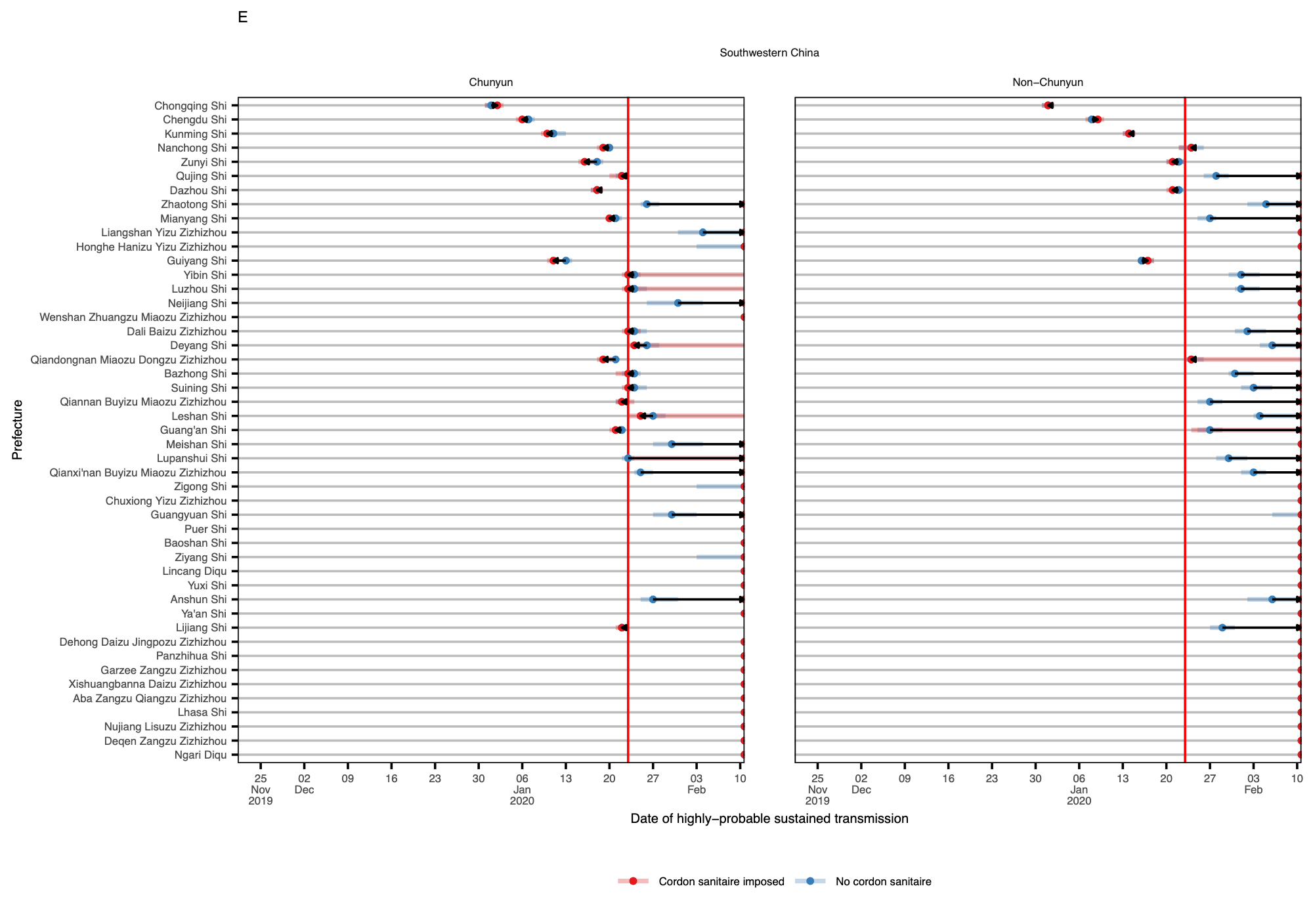

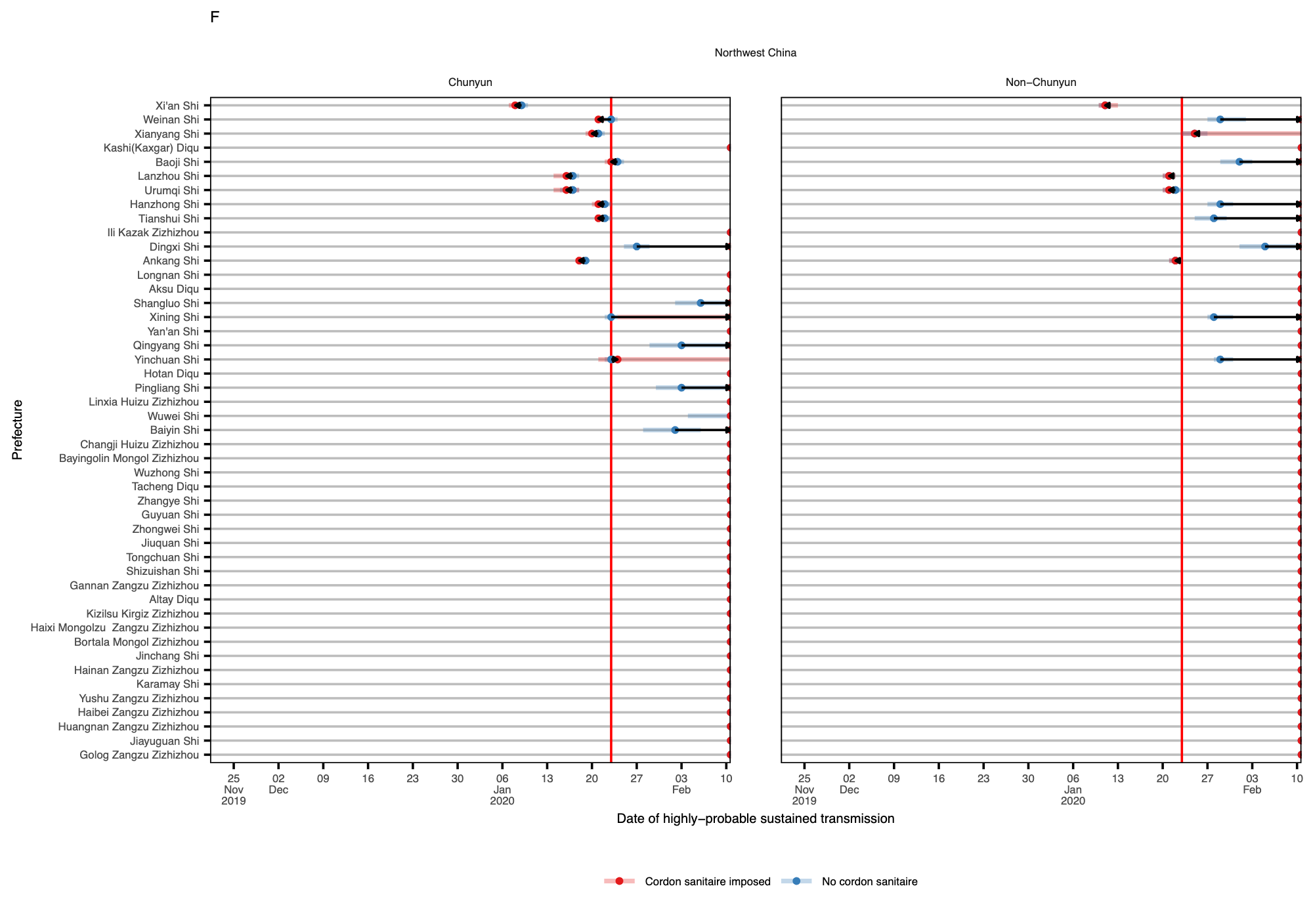
Figure S1 - Date at which the mean probability of sustained transmission breaches 95%* [*[20]*](https://www.zotero.org/google-docs/?GufbYS) *for cordon sanitaire imposed (red) vs no cordon sanitaire (blue) for Chunyun (left panel) and Non-Chunyun (right panel) travel patterns for each prefecture given travel patterns from Wuhan. Red vertical line indicates the date the cordon sanitaire was imposed. Black lines with arrows indicate time difference between scenarios; arrows pointing right indicate delay, arrows pointing left indicate advance. Points on the right limit of the graph indicate that no outbreak has occurred by that date. Outbreak probability calculated with R0=2.2 and k=0.1. Prefectures sorted by population. Prefectures grouped into six regions of China based on the first digit of the administrative unit code. A = Northern China, B = Northeast China, C = Eastern China, D = Central and Southern China, E = Southwestern China, F = Northwest China.*


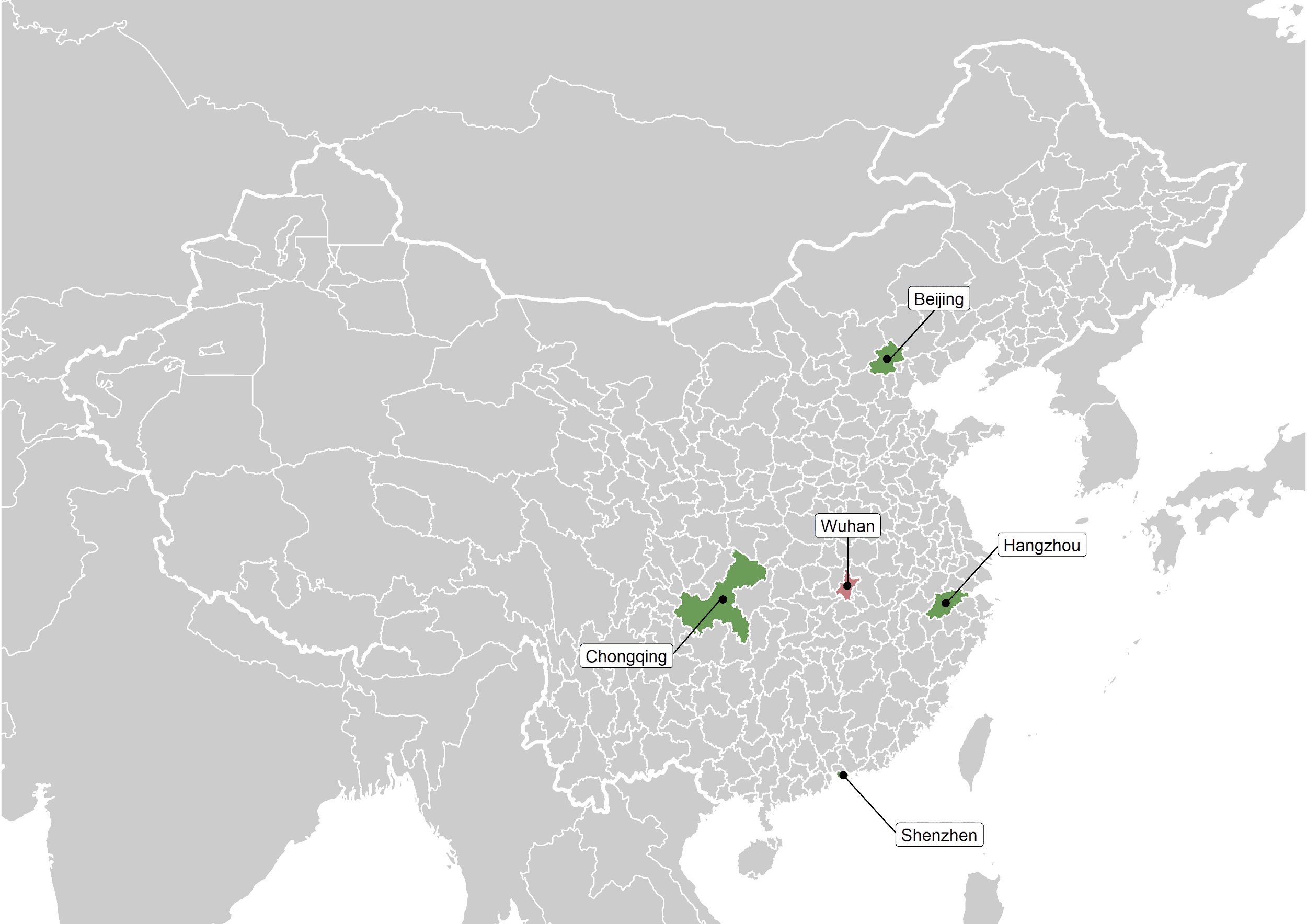


*Figure S2 - Location of Wuhan (centre, pink) and the four cities of interest (green) in mainland China.*

*
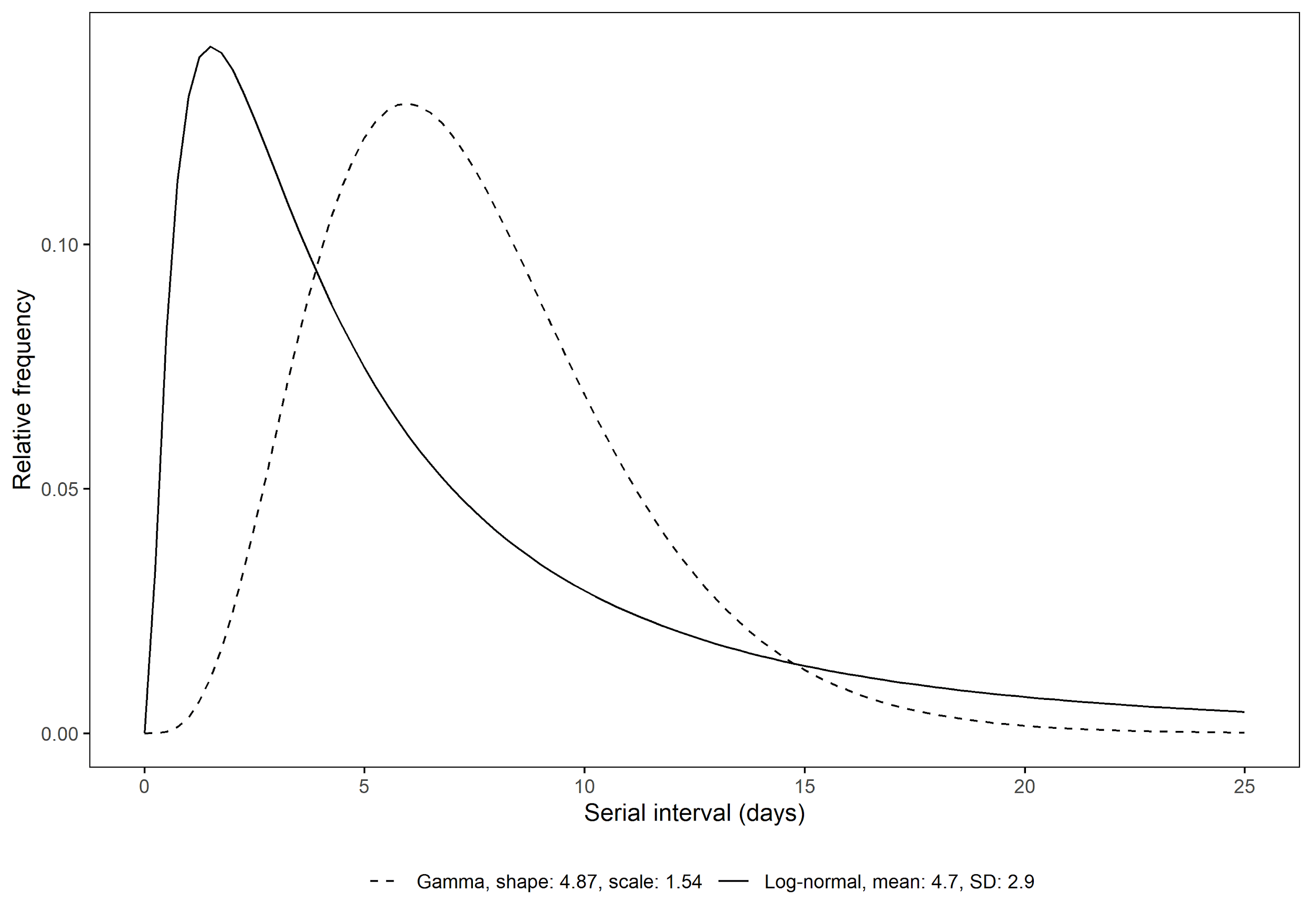
*

*Figure S3 - Delay distributions for the serial interval of COVID-19 infection from literature. Log-normal with mean 4.7 days and standard deviation of 2.9 days* [*[18]*](https://www.zotero.org/google-docs/?PUWEgO) *and a Gamma with mean 7.5 days and standard deviation of 3.4 days* [*[19]*](https://www.zotero.org/google-docs/?YdNbQt) *(converted to shape = 4.87 and scale = 1.54 using epitrix R package* [*[22]*](https://www.zotero.org/google-docs/?iYWnds)*).*

*
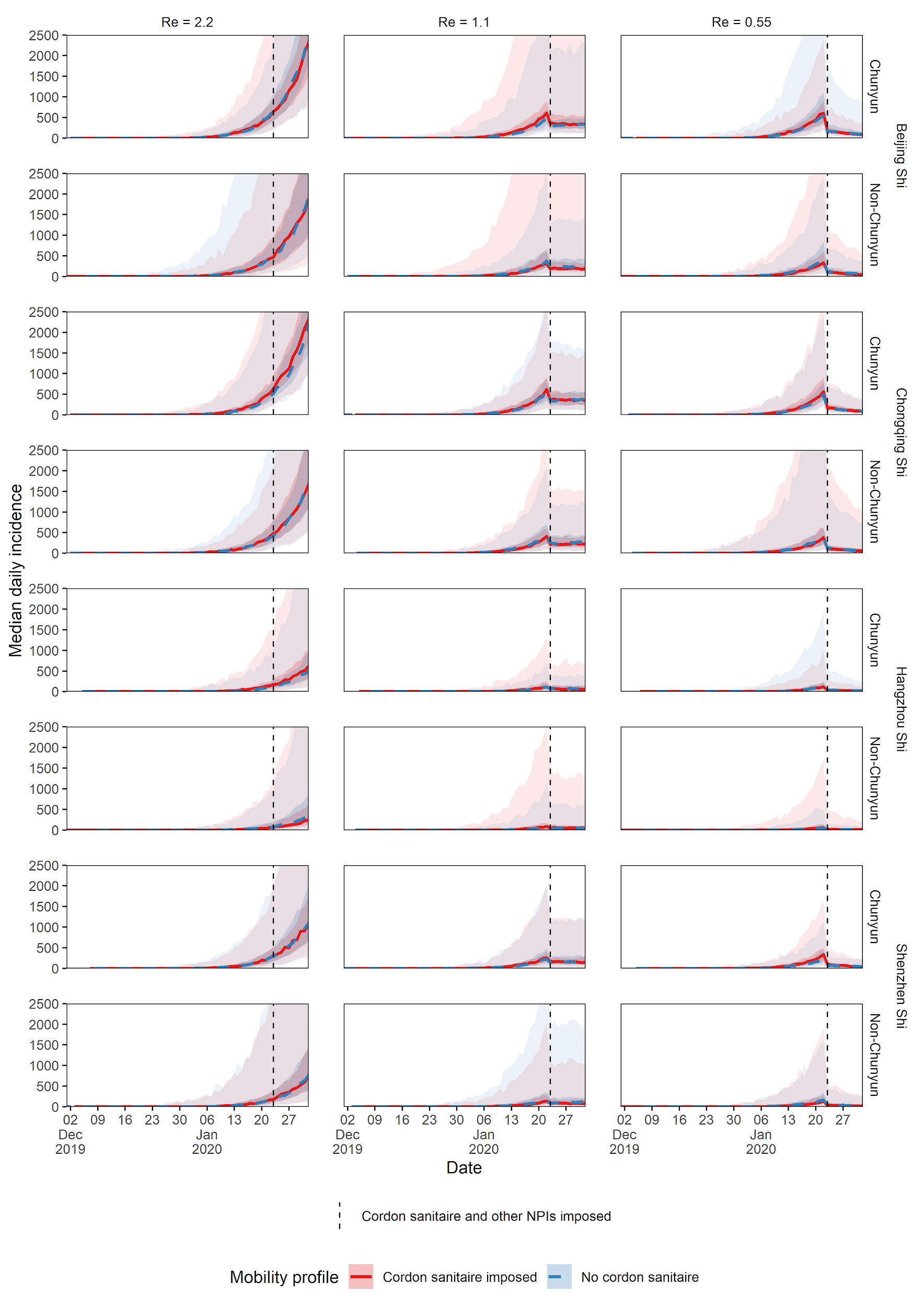
*

*Figure S4 - Median daily incidence of COVID-19 (log-scale, shaded areas indicate 50% and 95% confidence intervals) in the four cities of interest, for Chunyun vs. Non-Chunyun, cordon sanitaire imposed (red, solid) vs. no cordon sanitaire (blue, dashed), and for varying values of the effective reproduction number R_e_, where R_e_ = 2.2 (no change, unmitigated local outbreak), reduced from 2.2 by 50% to 1.1 (mitigation of outbreak, R_e_>1), and 75% to 0.55 (suppression of outbreak, R_e_<1).*

*
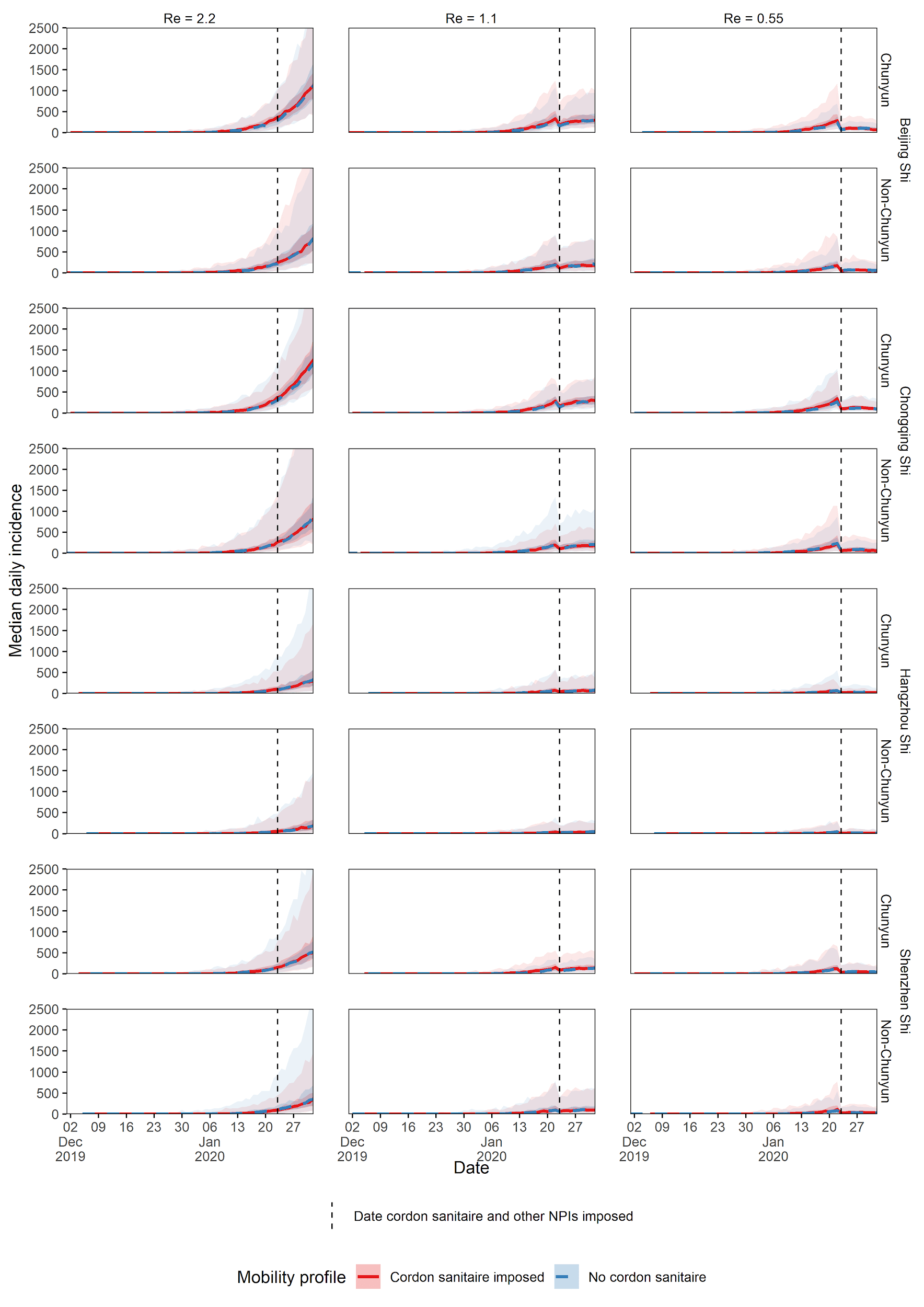
*

*Figure S5 - Alternative serial interval of mean 7.5 days (SD: 3.4). Median daily incidence of COVID-19 (shaded areas indicate 50% and 95% confidence intervals) in the four cities of interest, for Chunyun vs. Non-Chunyun, cordon sanitaire imposed (red, solid) vs. no cordon sanitaire (blue, dashed), and for varying values of the effective reproduction number Re, where Re = 2.2 (no change, unmitigated local outbreak), reduced from 2.2 by 50% to 1.1 (mitigation of outbreak, Re>1), and 75% to 0.55 (suppression of outbreak, Re<1).*

*
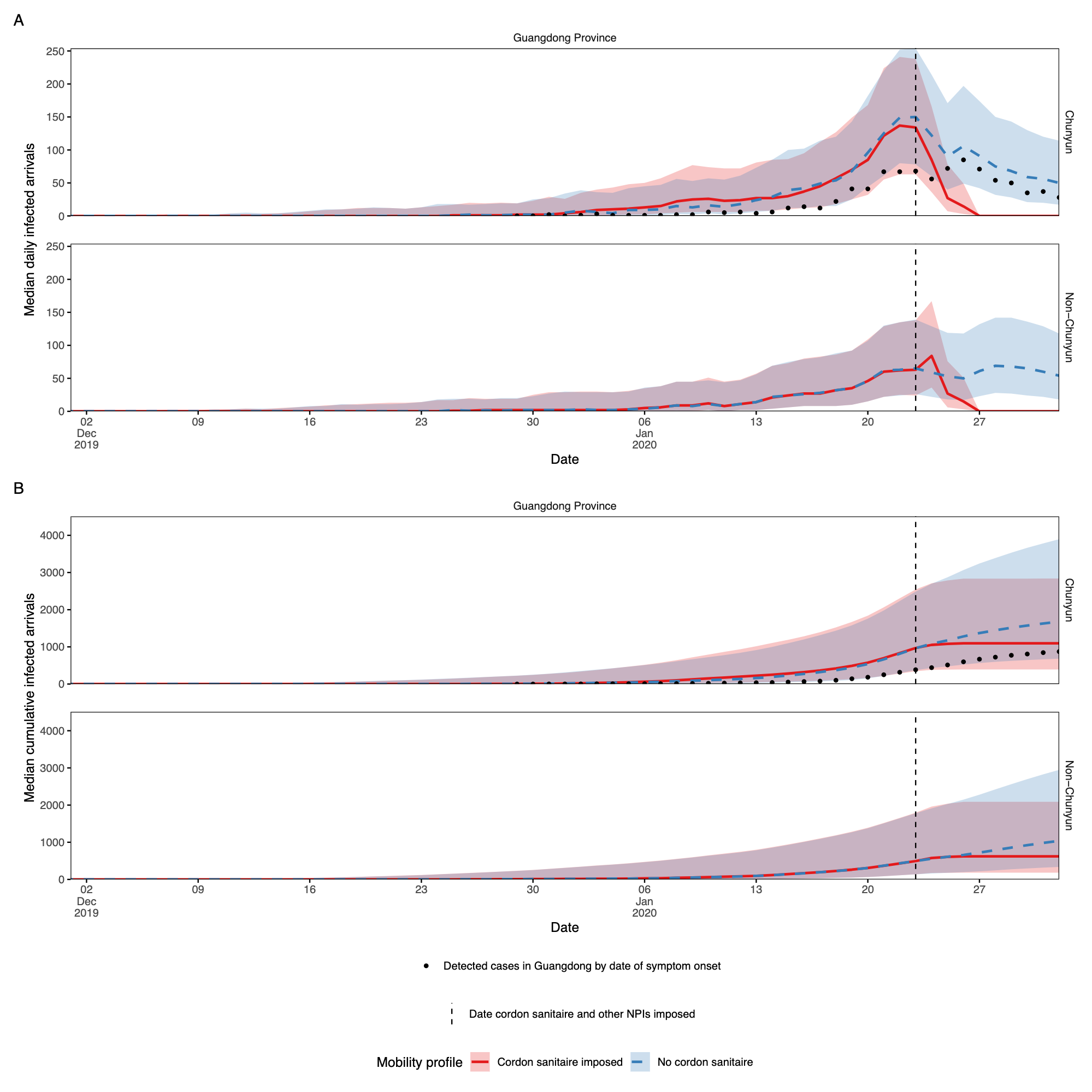
*

*Figure S6 - Simulated infected arrivals to Guangdong province from Wuhan (median and 95% UI). A. Estimated median number of daily infected arrivals and B. Estimated cumulative number of infected arrivals from Wuhan for Chunyun vs. Non-Chunyun, and cordon sanitaire imposed (red, solid) vs no cordon sanitaire (blue, dashed). Shaded area indicates the 95% uncertainty interval. Vertical dashed line indicates the date the cordon sanitaire was imposed. In Scenario 1, infected arrivals appear to follow a similar rising and falling trend as the reported imported cases in Lu et al. (2020), albeit with a lag of several days* [*[26]*](https://www.zotero.org/google-docs/?712ra7) *(black dotted line).*

*
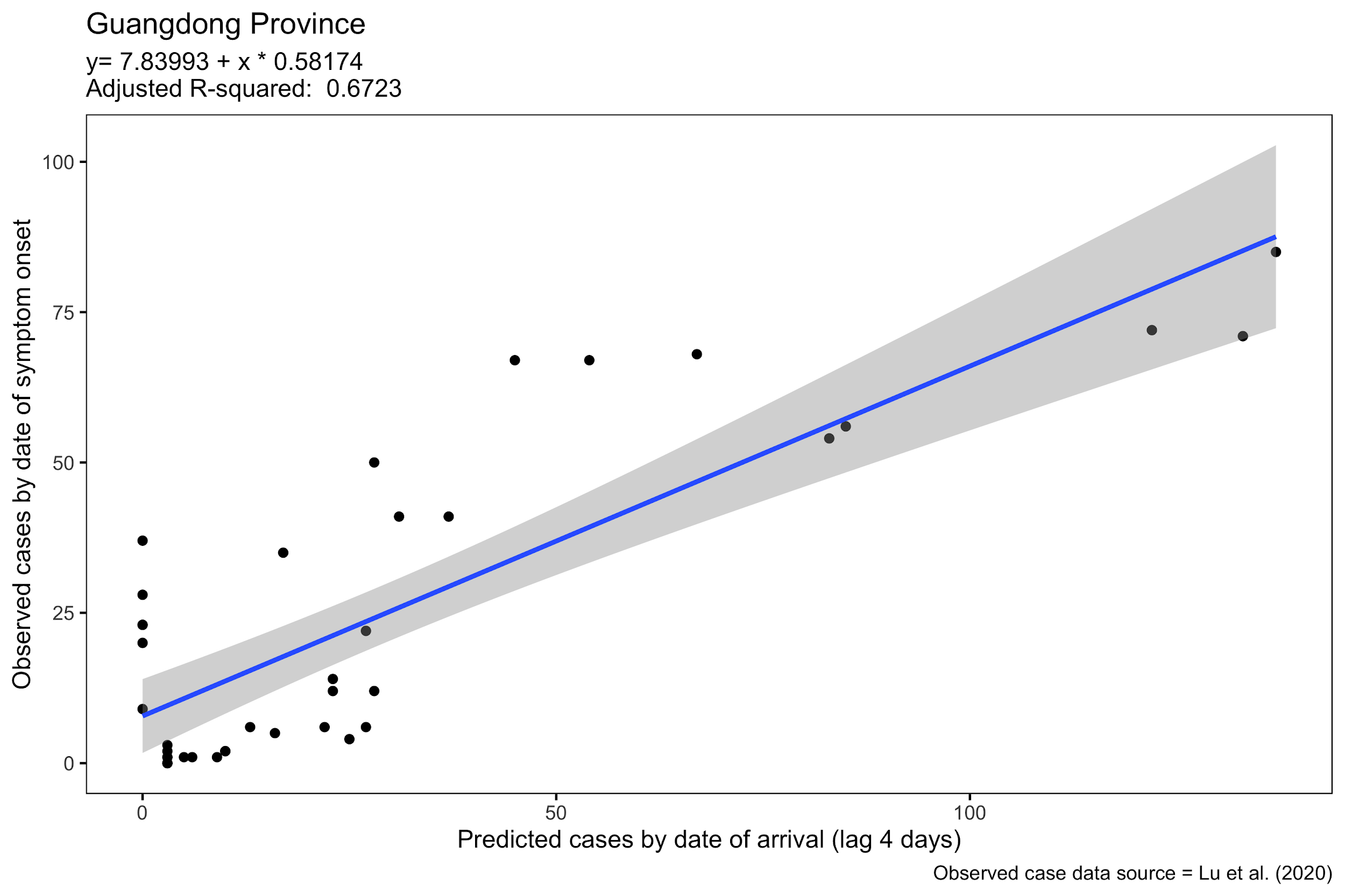
*

*Figure S7 - Observed imported cases by date of symptom onset vs. predicted imported cases by date of arrival with a lag of 4 days.*
